# Potential and perspectives of halide perovskites in light emitting devices

**DOI:** 10.1186/s40580-023-00395-1

**Published:** 2023-10-13

**Authors:** Khan Lê, Niusha Heshmati, Sanjay Mathur

**Affiliations:** Institute of Inorganic Chemistry, University of Cologne, Greinstraße 6, 50939 Cologne, Germany

**Keywords:** Halide perovskite-based LEDs (PeLEDs), PeLEDs structure, Stability & scalability, Device challenges, Perovskite nanocrystals, Colloidal perovskite, Electroluminescence

## Abstract

Light emitting diodes (LEDs) have become part of numerous electrical and electronic systems such as lighting, displays, status indicator lamps and wearable electronics. Owing to their excellent optoelectronic properties and deposition via simple solution process, metal halide perovskites possess unique potential for developing halide perovskite-based LEDs (PeLEDs) with superior photoluminescence efficiencies leading to external quantum efficiencies beyond 20% for PeLEDS. However, the limited durability, high operative voltages, and challenges of scale-up are persisting barriers in achieving required technology readiness levels. To build up the existing knowledge and raise the device performance this review provides a state-of-the-art study on the properties, film and device fabrication, efficiency, and stability of PeLEDs. In terms of commercialization, PeLEDs need to overcome materials and device challenges including stability, ion migration, phase segregation, and joule heating, which are discussed in this review. We hope, discussions about the strategies to overcome the stability issues and enhancement the materials intrinsic properties towards development more stable and efficient optoelectronic devices can pave the way for scalability and cost-effective production of PeLEDs.

**Graphical Abstract:**

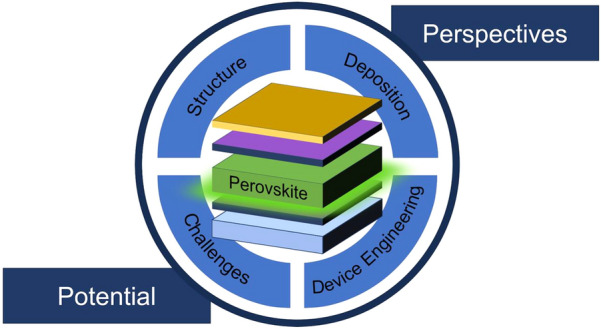

## Introduction

Light emitting diodes (LEDs) are widely utilized in displays, ambient lighting, signaling, gadgets and many other consumer electronics due to their high color purity and narrow light emission wavelengths. The rise of LEDs is attributed to several major advantages over traditional, incandescent light sources, for instance, LEDs can be switched between the ‘on’ and ‘off’ states quickly, they are more efficient as less heat is produced during operation and they exhibit high chromaticity [1]. Hybrid organic–inorganic and all-inorganic lead halide perovskites (APbX_3_) have emerged as promising photoactive medium for solid-state lighting and visible light communication, leading to significant progress towards highly efficient photovoltaic technologies. However, the high crystallinity and poor substrate coverage in solution-processed perovskite absorber causes high operating voltage and low luminescence efficiency, thus hindering their practical applications.

**Fig. 1:**
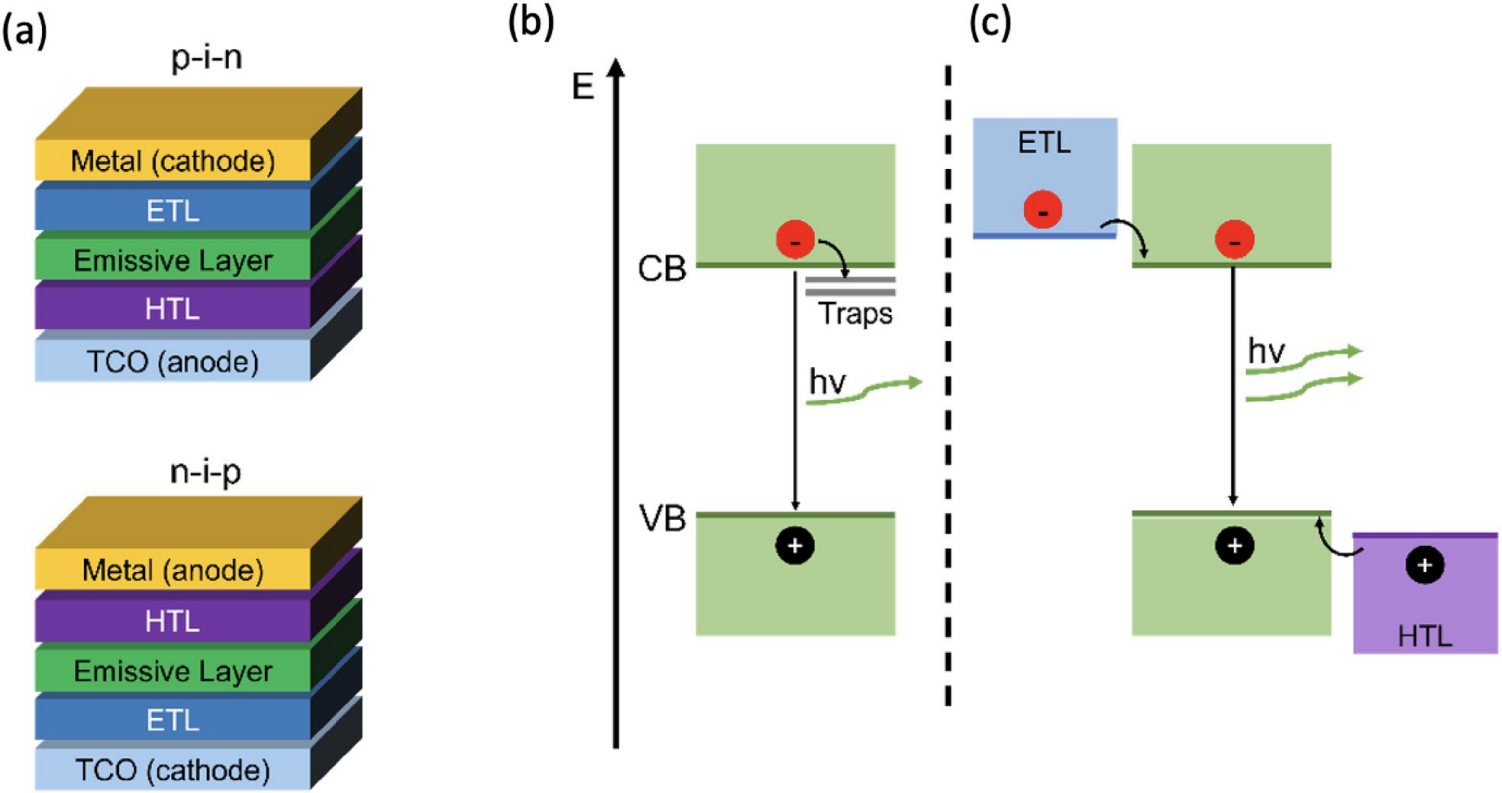
**a** Schematic of simple device stacks in n-i-p and p-i-n configuration (**b**, **c**) Charge carrier recombination mechanism in perovskite (**b**) Band diagram showing trap-assisted non-radiative recombination and radiative recombination pathways and (**c**) the ideal case with radiative recombination only

The first perovskite (ABX_3_) solar cells (PSCs) were based on dye-sensitized solar cells, whereas the first PeLEDs had a strong interconnection with the organic LED research. A variety of organic hole and electron selective layers are thus common in leading research groups to tun the devices towards high efficiency in a concerted manner [2]. The injection and transporting layers are selected to efficiently transfer charge carriers into the emitting material at a similar rate to improve external quantum efficiency (EQE), roll-off and lifetime [3]. The injection and transporting layers are not inherently necessary as proven by “single-layer” light emitting devices [4, 5]. In such single-layer devices, the ion migration under applied voltage creates a homojunction within the perovskite through charge accumulation/self-doping. The ambient and temperature stability of all-inorganic perovskites (e.g., CsPbBr_3_) is usually higher than that of organic–inorganic hybrid perovskites (e.g., CH_3_NH_3_PbBr_3_). The narrow emission bandwidth and the wide color gamut of perovskites make them especially interesting for solid-state display technology [6]. The most common LED devices are based on multilayered structures with the emissive layer sandwiched between charge selective layers, deposited on glass or plastic substrates with a transparent conductive oxide like indium-doped tin oxide (ITO) or fluorine-doped tin oxide (FTO) as bottom electrode and a metal as top electrode. Thin film LEDs with organic emitters, quantum dots or perovskite emitter can be built with a normal or inverted architecture, depending on whether the TCO is used as anode (injection of holes) or cathode (injection of electrons) (Fig. 1a) [7].

An optimized charge carrier transport is achieved in the presence of hole injection layer (HIL) and hole transporting layer (HTL) as well as electron injection layer (EIL) and electron transporting layer (ETL). The charge carriers eventually reach the emissive layer (EL) where they form excitons and can recombine radiatively [8]. In normal (p-i-n) stacked LEDs, electrons must be injected into the conduction band/lowest unoccupied molecular orbital (LUMO) of the ETL, hence low work function metals like Ba, Ca, LiF/Al or Ag are generally chosen as cathode. For flexible devices, single walled carbon nanotubes (SWCNTs) or polyethylene-terephthalate (PET)/ITO as substrate and silver nanowires (Ag NWs) as top electrode were implemented [9, 10]. The first room temperature perovskite bright light emitting diodes (PeLEDs) were reported by Tan et al. [1], when they demonstrated near-infrared (CH_3_NH_3_PbI_3–*x*_Cl_*x*_), red (CH_3_NH_3_PbBr_2_I) and green (CH_3_NH_3_PbBr_3_) perovskite LEDs*.* Following the impressive success of perovskite solar cells, researchers enhanced the efficiency of perovskite LEDs from 0.1% to 23% (red emitting) in only 7 years [11]. Compared to all-inorganic III-V / II-VI semiconductor-based LEDs (e.g. GaN, InP, ZnSe, CdSe; EQE: 27.6%, Luminance: ~ 100,000 cd m^−2^) [12] and organic light emitting diodes (OLEDs, EQE: 41%, Luminance: ~ 200,000 cd m^−2^) [13] the efficiencies and luminance of PeLEDs (EQE: 23%, Luminance: ~ 150,000 cd m^−2^) [11, 14] have reached to comparable levels through consistent materials engineering. The narrow full width at half maximum (FWHM) of 5–40 nm and 140% coverage of the national television systems committee (NTSC) standard color range make PeLEDs exceptionally suited for high-quality displays [15]. The persisting major downside of PeLEDs is their operational lifetime in comparison to the conventional and organic LEDs that last for over 10,000 h, while the half-life time of PeLEDs only spans a few minutes to hours with a maximum of 250 h achieved by Wang et al. [16–18] Thus, the main challenge for PeLED research is the prolongation of the device operation lifetime, as even the most enduring devices’ lifetimes cannot compete with the traditional semiconductor LEDs (~ 50,000 h) and OLEDs (blue: 11,000 h; green: 400,000 h, red: 200,000 h) [19–22].

Hybrid organic–inorganic halide perovskites represent a new generation of promising and cost-effective materials for photovoltaic and light emitting devices. The extensive efforts carried out world-wide (> 70,000 original publications) in developing the science and technology of perovskite-based solar cells and related opto-electronic devices has unequivocally demonstrated their potential for commercial breakthroughs [23] (Our road map publication: APL Mater. 2021, 9, 109202). The integration of perovskites in devices is now at a stage where their techno-economic potential is being evaluated on higher technology readiness levels while still many fundamental questions are being unraveled. The demonstrated feasibility to use a variety of scalable solution-based printing and coating technology to make high performance perovskite devices offer a leverage over the first-generation of photovoltaic devices based on silicon and compound semiconductors (Cu(In,Ga)Se_2_, CdTe, etc.). Indeed perovskite solar cells are the best solution-processed solar cell technology to date with certified device performance (PCE) of > 25.7% [24].

This review focuses on the current state-of-the-art processing of all-inorganic and organic–inorganic hybrid perovskites for optoelectronic application with thrust of light-emitting devices. We highlight the recent challenges and milestones towards stable and highly efficient perovskite devices and discuss prospects for compositional and defect engineering of perovskites with focus on strategies for the enhancement of desirable properties that go beyond the configurational entropy approaches and iterations.

## Perovskite structure

**Fig. 2:**
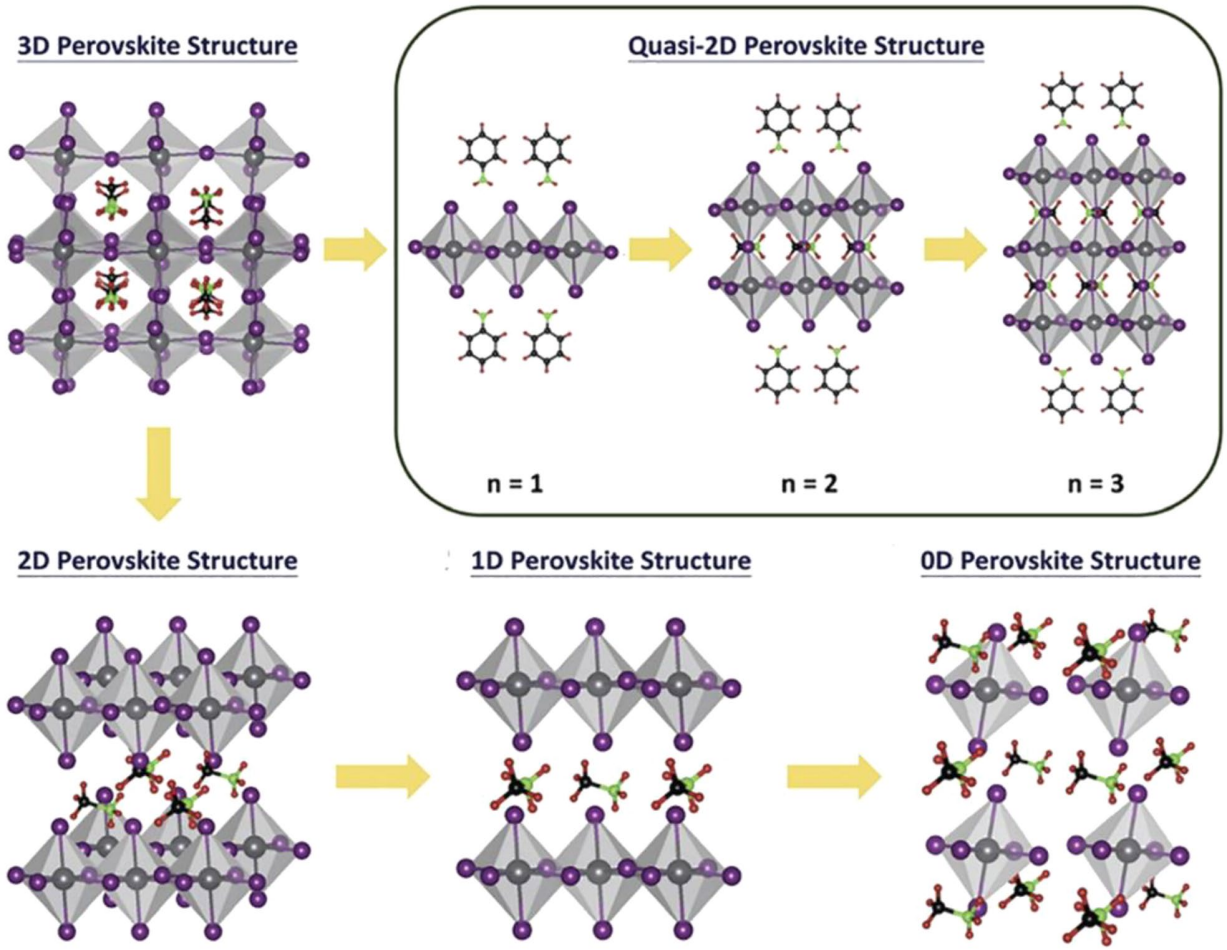
Schematic of perovskite materials with different structural dimensionality (Reprinted from Ref. [27] with permission from the Royal Society of Chemistry)

Perovskites represent an important class of crystalline compounds based on the structure of the calcium titanate (CaTiO_3_) mineral, which was discovered in 1839 by Gustav Rose and named after the mineralogist Lew Alexejewitsch Perovski [25]. The general chemical structure can be simplified into ABX_3_, where A and B sites are occupied by metal cations and X positions are occupied by anions such as oxides or halides. The B-site cation is coordinated by the anions to form octahedra, while the A-sites consists of 12-coordinated cations [26]. Each octahedron is connected through bridging X-atoms leading to a three-dimensional network. This connectivity can vary depending on the ionic size of the components to give lower dimensional frameworks e.g., formation of a layered 2D perovskite structure for larger organic cations on the A-site (Fig. 2).

**Fig. 3:**
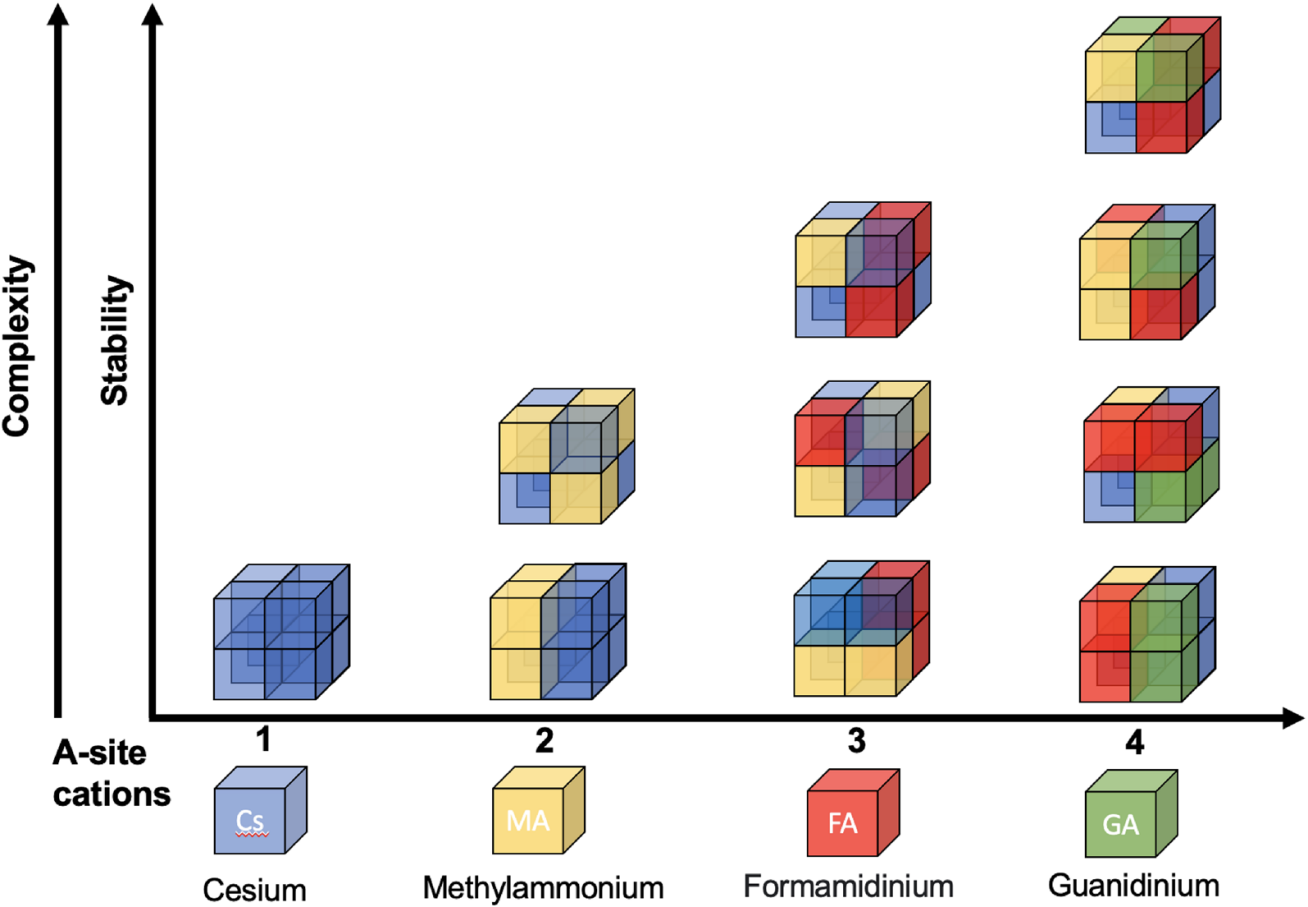
Possible compositional entropy in organic–inorganic hybrid perovskites and the related improvement in efficiency and stability of PV and PeLED devices

Oxide perovskites (ABO_3_) exhibit ferroelectricity, superconductivity, good electrical conductivity, and high catalytic activity [28], which makes this class of crystalline material indispensable for functional applications. Furthermore, the importance of the perovskite structure arises due to its compositional flexibility that allows facile intermixing of suitable cation- or anion combinations at the A-site (A^1^_1−x_A^2^_x_BX_3_), B-site (AB^1^_1−x_B^2^_x_X_3_), X-site (ABX^1^_3−x_X^2^_x_) or even at all three possible sites at once (A^1^_1−x_A^2^_x_B^1^_1−y_B^2^_y_X^1^_3−z_X^2^_z_), resulting in different properties (e.g. band structure, band gap, absorption, photoluminescence, ferroelectricity). This flexibility is dominant especially in organic–inorganic hybrid halide perovskites, where the included metal usually belongs to the 14th group such as tin (Sn^2+^) and lead (Pb^2+^) occupying the B-site and the halide anions frequently vary between chloride (Cl^−^), bromide (Br^−^) and iodide (I^−^) occupying the X-site. Thereby the BX_6_ octahedra form a corner sharing network in which monovalent cations such as CH_3_NH_3_^+^ (MA), NH_2_CH=NH_2_^+^ (FA) and Cs^+^ occupy the A-site to compensate the total charge and to stabilize the lattice [26] (Fig. 3).

The crystallographic stability of perovskites can be predicted by an empirical *Goldschmidt* tolerance factor *t* (Eq. 1) [29], which considers that the radius of each component *R*_*A*_, *R*_*B*_ and *R*_*x*_ can be described by a densely packed cubic structure with a tolerance factor of *t* = *1*.

1 $$t = \frac{{\left( {R_{A} + R_{X} } \right)}}{{\surd 2 \cdot \left( {R_{B} + R_{X} } \right)}}$$

The Goldschmidt rule provides fundamental guidelines to predict stable perovskite structures. Since the phase-transitions are strongly related to thermodynamical parameters, the compositional variations or configurational entropy plays a critical role in determining the structure–property-stability relationship and in designing new perovskites as well as in predicting their properties.

For halide perovskites (X = F, Cl, Br, I), the tolerance factor varies in between 0.81 < *t* < 1.11 [30]. If *t* lies in the narrower range of 0.89 < *t* < 1.0 [31], the cubic structure is likely to be stabilized, whereas tetragonal or orthorhombic structures are formed at 0.71 < *t* < 0.9 [31] and higher *t* > 1 [31] result in hexagonal non-perovskite structures that are not photoactive. Temperature changes can lead to phase transitions for example the transition to the more symmetric perovskite structure of MAPbI_3_ with the cubic α-phase occurring above 327.4 K [32, 33].

**Fig. 4:**
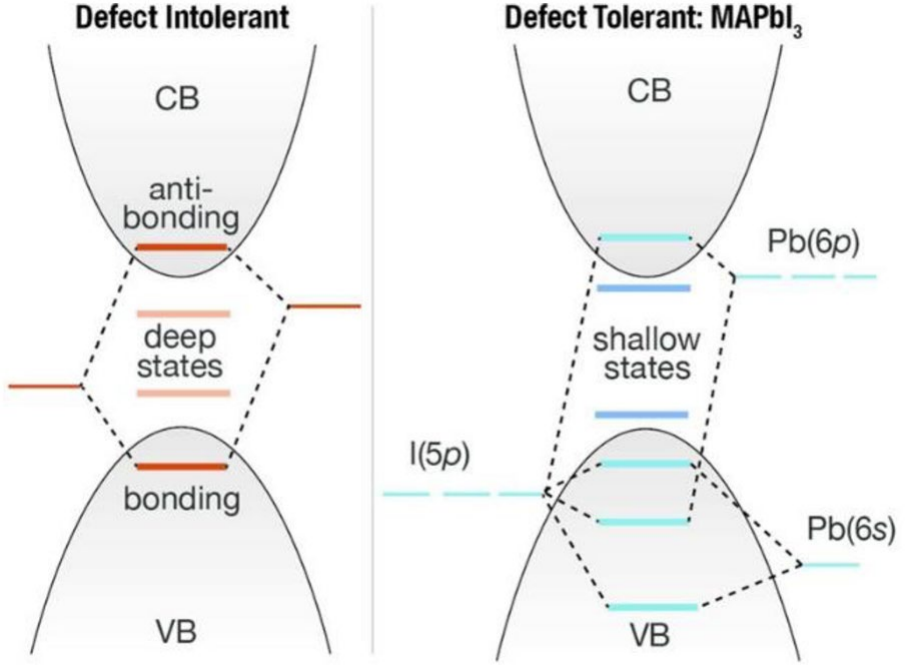
Band structure and trap states comparison between conventional (III–V or II–VI) semiconductor materials to MAPbI_3_ (Reprinted with permission from Ref. [34]. Copyright © 2017 American Chemical Society)

The metal-halide framework strongly directs the optoelectronic properties, since the valence band consist of the s^2^ lone pair of Pb^2+^ and halide p-orbitals, and the conduction band consists of lead 6p and halide p-orbitals [35]. The metal-halide framework connectivity and geometry can be directed by the A-site cations through steric and coulombic interactions causing an octahedral tilting which is responsible for the changes in the electronic structure near the band edges. Lead halide perovskites have a low formation energy, providing facile growth [36]. The high defect tolerance with immunity to anti-site and interstitial point defects compared to conventional semiconductors like GaAs enables processing from abundant, lower purity starting materials (Fig. 4) [34]. Furthermore, in combination with the highly ionic structure, low trap densities and low exciton binding energies, the defect tolerance results in long carrier diffusion lengths, making the perovskite an excellent choice for solar cells [37–39]. For light emitting diodes, however, the carriers should be confined to efficiently recombine radiatively [40]. This confinement can be achieved by size reduction, as for nanocrystals the quantum confinement increases the radiative recombination and thus lead to higher PLQY [41]. The nanocrystals are usually prepared as colloidal solution before application on chosen device substrates, however the perovskite nanocrystals can also be prepared in situ by means of nanocrystal pinning (NCP), a procedure where solvents or additives are exploited to check the grain growth in order to keep the perovskite crystals small. Park et al. found due to the defect healing effect NCP can improve the radiative recombination by decreasing the effective defect density in grain boundaries [42, 43]. Furthermore, reduction of defects can improve optoelectronic properties and a lower defect density can be achieved either by compositional engineering or by optimizing the device processing steps [44–46].

## Perovskite deposition

Preparation and optimization of perovskite thin films is exhaustively studied in the last 11 years due to solar cell research that has prepared the science and technology of perovskite-based solar cells and related opto-electronic devices twoards potential commercial breakthroughs [47, 48]. The characteristic device properties such as suitable and tunable band gap [49], long charge-carrier lifetimes [50], relatively low recombination activity at the interfaces, high mobilities of electrons and holes, and high defect-tolerance, have produced highly efficient photovoltaic devices [51]. Accordingly, one of the superior properties of organic–inorganic perovskites that makes them potent material for manufacturing photovoltaic devices is the possibility of low-temperature deposition [52]. Quality of perovskite thin films acts a crucial role in a device’s performance, while the figure of merit depends on the method chosen to fabricate the perovskites absorber films under the consideration of cost effectiveness (solution processing vs. evaporation techniques) and scale-up potential [53]. Recently, the EQE for PeLEDs have exceeded 20% for laboratory scale devices (< 0.1 cm^2^) [27]. However, producing highly crystalline and uniform perovskite film is still remains a demanding task because of diverse phenomena that occur during film formation in particular for large-area PeLEDs (≥ 1 cm^2^) and are detrimental for optoelectronic properties [27, 52]. As depicted in (Fig. 2) structural dimensionality of perovskite materials including 3D perovskites, 2D/quasi-2D perovskites, and 1D nanocrystals/quantum dot perovskites are commonly used as emitter layer in PeLEDs [54]. Generally, in 3D perovskites trap-assisted nonradiative recombination is a limiting factor and reducing the size of perovskite platelets has direct effect on the increase of PLQE [55].

## Morphology and crystallinity

**Fig. 5:**
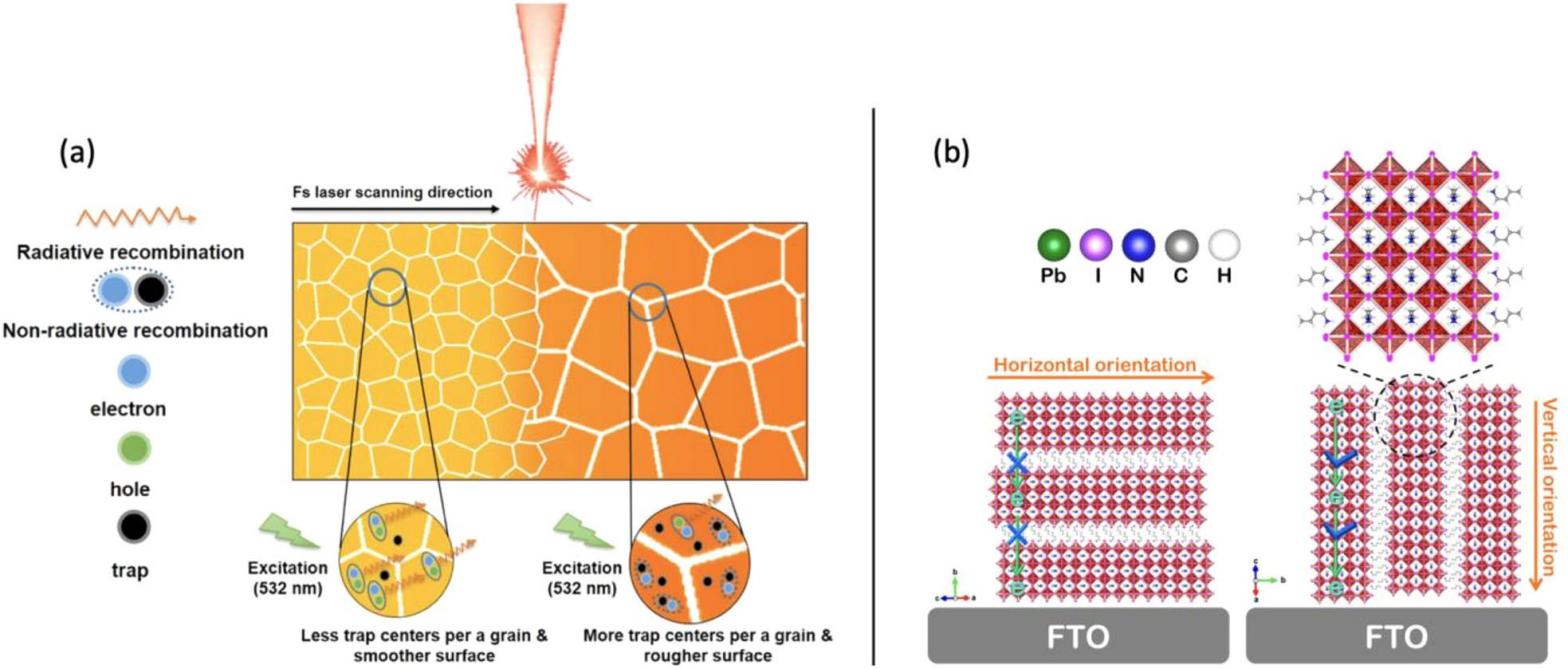
Schematic illustration of (**a**) the fs laser-processed perovskite film and the effect of smaller grain size on enhanced radiative recombination [58]. Copyright © 2023, American Chemical Society. b Effect of horizontal and vertical orientations relative to FTO substrate on charge transfer [59]. Copyright ® 2019, American Chemical Society

The morphology, surface roughness, and crystallinity of perovskite thin films are deterministic parameters influencing the efficiencies of perovskite devices because an interplay of these parameters noticeably affect optical and electrical properties. Hence, uniform, pinhole-free, and dense enough perovskite thin films are necessary to reduce the structural defects and ensure stable and durable operation of optoelectronic devices [52]. An uncontrolled formation of shunt paths and pinholes possibly results from inhomogeneous film deposition resulting in poor surface coverage of perovskite layer, consequently leading to serious problems in the fabrication of high efficiency PeLEDs [55]. Although large grain sizes in perovskite layer are preferred for better charge transport and highly efficient PSCs, grain size reduction can spatially confine charge carriers and elevate radiative recombination, which leads to enhancement in photoluminescence (PL) intensity [56, 57]. Therefore, Chen et al. utilized femtosecond (fs) laser processing to reduce the grains size and the number of trap centers per grain (Fig. 5a) [58]. It is also beneficial to achieve favorable crystallographic orientations and anisotropy in perovskite grains for enabling carrier transport perpendicular to the substrate that is responsible for efficient charge extraction in the external circuit. This is particularly the case for 2D and quasi-2D perovskite films in which the metal halide perovskite layers should ideally orient perpendicular to the substrate. In fact, charge carriers can transport through the inorganic layers much easier than the transport via organic cations, and it can improve the charge mobility in 2D oriented perovskite films (Fig. 5b) [59, 60].

### Three-dimensional bulk perovskites

**Fig. 6:**
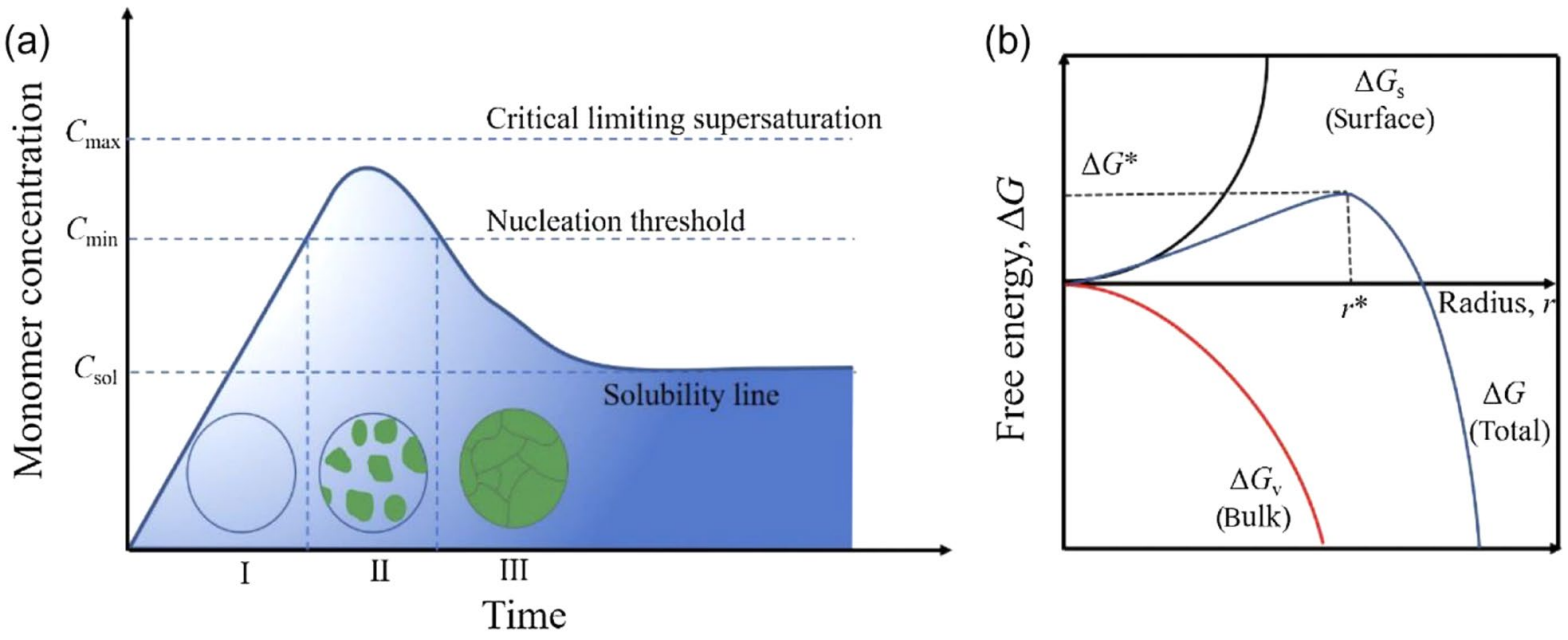
**a** Nucleation and growth mechanism of perovskite layer, **b** free energy diagram of uniform nucleation. This figure has been published in CCS Chemistry 2022; Droplet Manipulation and Crystallization Regulation in Inkjet-Printed Perovskite Film Formation is available online at 10.31635/ccschem.022.202101583 [65]. Copyright © 2022 Chinese Chemical Society

One of the necessary challenges that should be addressed is an understanding the role of dimensionality on the perovskite properties. One-step application of the perovskite thin films from colloidal perovskite inks make 3D bulk (lattice scale) perovskites fabrication outstandingly cost-effective and viable for large-scale device fabrication [53]. The solution formation mechanism (Fig. 6) of perovskite crystals is similar to nucleation of any other solid from a homogeneous solution that typically includes three stages of the solution reaching a supersaturation stage surpassing the critical saturation limit that leads to spontaneous nucleation of several ultrafine clusters (embryonic crystals) followed by subsequent growth and ripening processes that leads to thermodynamically stable larger crystals with reduced surface area and negative Gibbs free energy [53]. Initial studies on PeLEDs were performed based on 3D bulk films of perovskite [61]. Several experiments have been conducted based on polycrystalline bulk perovskite thin films in past 3 years to demonstrate a rapid progress that has led to EQEs values for 3D PeLEDs exhibiting more than 20% in both green and near-infrared regimes [62]. However, the low temperature crystallization makes the resulting material prone to defect formation and phase impurities that are detrimental for device performance [63]. Besides, the presence of perovskite phases in larger grain size range (100–500 nm) in the light emitting layer is an obstacle to get high quantum efficiency. Various processing methods such as antisolvent treatment, sequential deposition, acidic treatment, and vacuum evaporation are successfully utilized for addressing this problem and to tune the nucleation and growth process of perovskite crystals [53, 64].

### Two-dimensional perovskites

The compositional and structural versatility in halide perovskites allow to utilize large organic A-site cations (e.g., phenylbutyl-ammonium, phenylethyl-ammonium, *n*-butylammonium) [66] to create two-dimensional (2D) structures, consisting of layered perovskites with interlayers of organic cations [32]. Indeed, 2D perovskites emerged onto the optoelectronics stage as a promising emissive layer, with intrinsic advantages that have not been detected in their 3D bulk counterparts [67]. This includes high exciton binding energy around hundreds of millielectronvolts for 2D perovskites in comparison to 3D perovskites with tens of millielectronvolts, high PLQY (∼ 88%) for sky blue 2D perovskite LEDs fabricated by Sargent et al. [68]. The general formula of 2D-layered perovskites is L_2_A_n−1_PbnX_3n−1_ in which [PbX_6_]^4−^ octahedral units are separated by organic chain (L) barrier layers. The intermittent barrier layers help in confining the electrons and holes within the inorganic layers, leading to increased exciton binding energies and promoting radiative recombination rates, consequently 2D perovskites results in narrow emission width and high PLQY values [69]. For PeLED applications, the best studied perovskites are the one in which the A-site cations are butylammonium (BA) and phenylethyl ammonium (PEA) [69, 70]. Also, quasi-2D perovskite films that represent disordered mixed 2D-3D phases with a wide phase-width have gained considerable success in small-area PeLEDs due to their superior stability in comparison to the 3D counterparts, however the power conversion efficiency of devices based on quasi-2D perovskite materials still lags behind the efficiencies achieved with 3D perovskites [71]. Particularly, the quasi-2D perovskites have unique optical properties resulting from their different structural characteristics in comparison with conventional 3D and 2D perovskites [54]. Highly emissive films can be fabricated via spin-coating by precisely monitoring the perovskite crystallization process and solidification kinetics. Antisolvent assisted spin-coating has been proved as the most effective method to develop high-quality quasi-2D layers [72]. Since 2016 when the first research was reported, the field has witnessed quick development of quasi-2D perovskite optoelectronics especially in LED applications [54, 73]. However, achieving the desired and optimal 2D to 3D ratio in quasi-2D perovskites thin films is still iterative, and a precise control over the number of octahedral layers “n” in the target composition remains a synthetic challenge [74]. If the 2D:3D ratios are managed well, the emissive perovskite layer is passivated by the organic layer and less ion migration is observed, resulting in higher color purity and longer operational lifetimes for the LEDs [75, 76]. As a result, the use of 2D perovskites carries the potential to lead to more stable organic–inorganic hybrid PeLEDs. On the less desirable side, the bulky organic layer acts as an insulator and reduces the charge transport within the device, leading to lower EQE [77].

### Perovskite nanocrystals

**Fig. 7:**
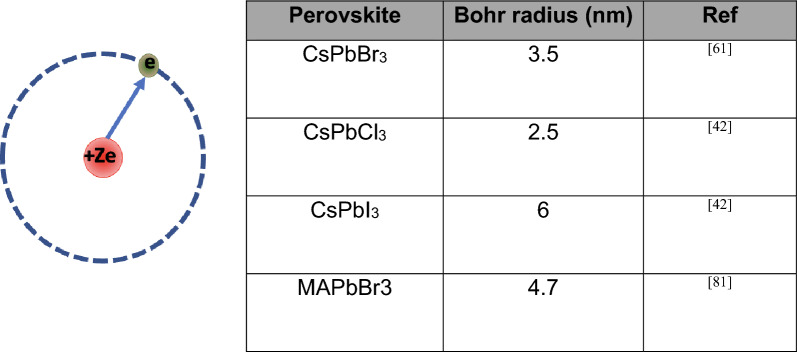
Bohr radius and calculated amounts for perovskites

Perovskite nanocrystals and quantum dots are considered exceptional light emitting materials because of their tunable size-dependent band gap, high color purity and broad absorption range. As a result, nanocrystals-based PeLEDs have displayed successful development since their first report by Song et al. in 2015 in which they utilized CsPbBr_3_ nanosheets to achieve 54% EQE [54, 78, 79]. In nanocrystals the quantum confinement increases the radiative recombination and thus lead to higher PLQY [80]. Moreover, the optical band gap and quantum effects in perovskite nanocrystals can be tailored by altering their composition and crystal size. In fact, for excitonic radiative recombination *Bohr* diameters as shown in (Fig. 7) must be bigger than the perovskite crystal size, and one possible approach to reach this goal is to use colloidal perovskite nanocrystals (NCs) [61, 81].

**Fig. 8:**
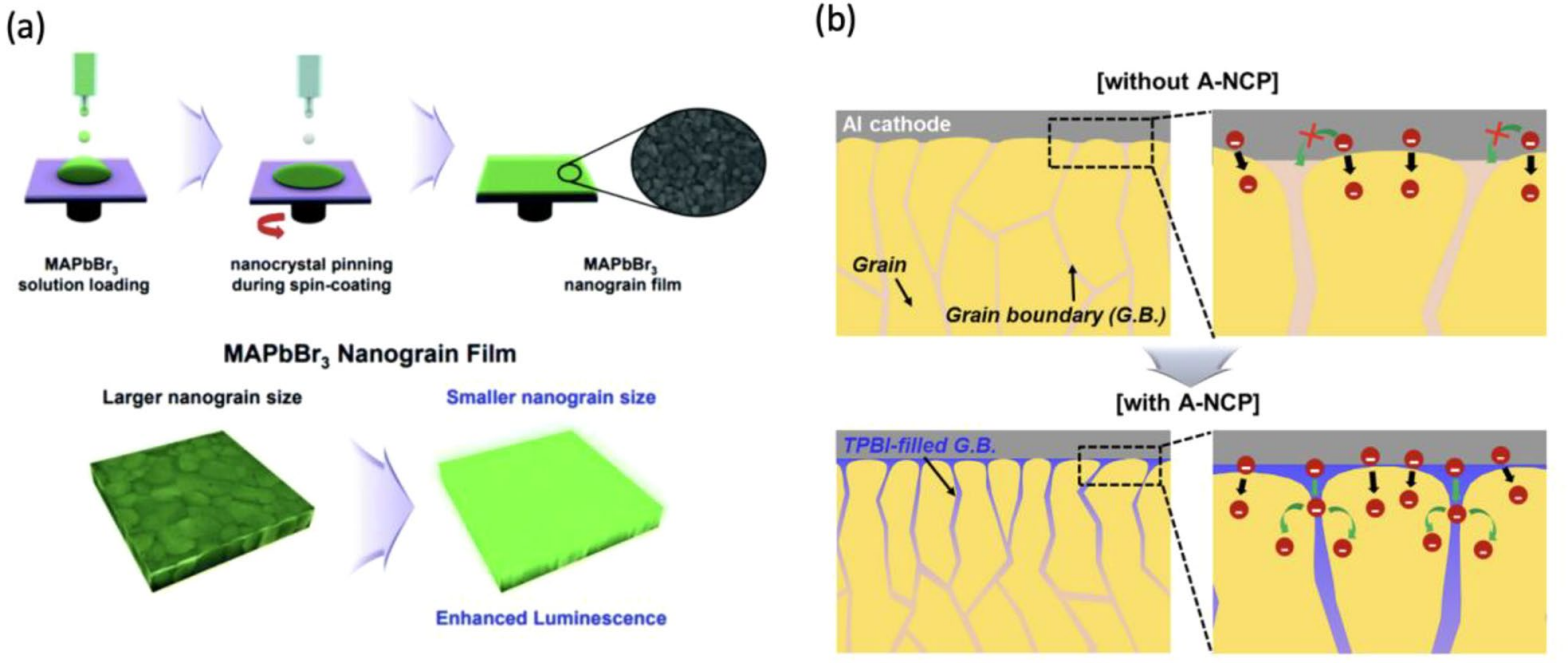
**a** Nanocrystal pinning method for perovskite emission property improvement. Reprinted from Ref. [82] Copyright © 2019, Nanoscale. **b** Schematic representation of the nanocrystal pinning by using anti-solvent during the spin-coating of perovskite inks [43]. © 2017 Elsevier Ltd. All rights reserved

Halide perovskite nanocrystals (NCs) are ideally suited for light-emitting applications due to their narrow emission bandwidths and high PLQE. Specifically, the family of inorganic CsPbX_3_ perovskites is the most promising class for application in light-emitting devices due to structural stability and lower defect denisties and their thin film device have showed high PLQE of > 85%. Nanocrystal pinning is a method, which uses antisolvent to pin the crystal growth on the substrate by flushing out the solvent used to prepare the perovskite inks. The approach of checking the grain boundary diffusion is appropriate for obtaining perovskite films composed of densely packed nanograins and has resulted in further improvement of the LED performance [82]. Adding a small organic molecule like TPBI to a bulk perovskite precursor ink leading to small perovskite grains embedded into organic shells in a spin-coating process, thereby mimicking the pre-synthesized nanocrystal structure while retaining the simplicity of film engineering and efficiency of bulk film application as shown in (Fig. 8) [43, 56].

One typical form of NCs is colloidal quantum dots (QDs), which emerged as favorable materials for high quality lighting technologies due to their superior properties such as solution-processability, color purity and tunability, and bright emission [83]. In 2015 Kovalenko and co-workers presented cube-shaped 4–15 nm edge-length CsPbX_3_ nanocrystals with PLQY up to 90% that were synthesized by the hot-injection method. The resulting particles are well defined and monodisperse with their final shape and size tunable by temperature and ligands [42]. Also, the morphology control can be utilized to enhance specific properties as shown by *Bi *et al*.* who reported PLQY and stability improvements of CsPbI_3_ by assembling nanowire clusters [84]. Another method for the synthesis of perovskite nanocrystals is the ligand assisted reprecipitation (LARP) which does not require dry solvents and inert conditions, however, this approach results in lower control over crystal size and size distribution, which leads to PLQY of 50–80% [85, 86]. As colloids, the perovskite nanocrystals have PLQY near unity, however application as thin films reduces the yield, and so far in thin films PLQY values of ~ 70% are reported [87]. The controlled synthesis, with the perovskite crystallization separated from the final device fabrication, results in more consistent device performance, as the crystallization of perovskites is known to be a critical step. Furthermore, the inherent small grain/crystallite size of nanocrystals is beneficial for quantum confinement and thus heightens PLQY and radiative recombination when defects are carefully managed [44, 45, 88].

While perovskite nanocrystals can yield the best values for photoluminescence, integration in devices is still problematic due to insufficient substrate coverage, film thickness and insulating stabilizing ligands. The additional step of nanocrystal synthesis is more complicated than a 3D perovskite ink and furthermore stabilizing ligands (oleyl amine, oleic acid) are implemented. Therefore, additional purification is necessary, and the ligands form an insulating layer over the light-emitting nanocrystal, affecting the charge carrier transport and thus device efficiency [89]. Another challenge is obtaining a sufficient film thickness with perovskite nanocrystals [90]. Accordingly, the device fabrication and performance of QDs PeLEDs need to be considerably amended before employing them towards commercialization.

### PeLEDs based on colloidal perovskite nanocrystals

PeLEDs prepared from colloidal perovskite nanocrystals have gained increased attention with concerted efforts being made by materials chemists and device engineers over the last 8 years. One of the intrinsic advantages of using low-dimensional perovskite nanocrystals relates to effective confinement of excitons, when compared to relatively low exciton bonding energy in perovskite films (~ 40 meV). The potential of colloidal perovskite nanocrystals for LED applications originates from their bright luminescence, which is however adversely affected by the limited stability and defect-rich surface of perovskite nanocrystals. As a result, various strategies for post-synthesis surface modification of perovskite nanocrystals have been developed, for instance, ligand attachment for surface stabilization or anion exchange to passivate surface defects. The main research aspects of colloidal routes to PeLEDs can be separated into three major areas: (1) crystal growth and size control, (2) surface stabilization and ligand engineering, (3) perovskite integration and device engineering [91].

The focus of phase-selective synthesis and crystal engineering lies in tailoring the synthesis strategies towards uniformly grown crystals with high crystallinity and low defect density. In addition, compositional engineering based on iso-valent substitution of perovskite-constituting ions and fabrication of core–shell structures to increase the structural and thermal stability of perovskite nanocrystals has witnessed a phenomenal advancement in the understanding of solution-based synthesis of perovskites [92–97]. However, the low stability of pristine nanocrystals and low electrical conductivity of ligand-capped perovskites crystals continues to hamper their device application and technological potential.

Besides the higher exciton binding energy, high-quality perovskite emitters demand effective and efficient surface functionalization strategy in order to passivate the surface defects and to achieve higher thermodynamic stability. The challenge of surface-engineering of perovskite nanocrystals is two-fold since strong binding between the perovskite crystals and surface ligands can hinder the charge injection and transport properties especially when using insulating ligands with long hydrocarbon chains (e.g., oleic acid, oleylamine) [98–100]. The quantum efficiency can be significantly improved by removing bulky hydrocarbon ligands with ionogenic molecules (e.g., quaternary ammonium salts). Given a strong interaction between the ionic ligands and surface-situated Pb atoms stable perovskite nanocrystals with enhanced stability and efficient charge injection properties can be obtained. In addition, a judicious ligand engineering during the synthesis of perovskite nanocrystals enable size and shape control and inhibit agglomeration of the colloidal nanocrystals. A number of recent studies in this field demonstrate the role of surface-conjugation with organic molecules in vacancy passivation, inducing self-assembly or for enhanced electronic coupling [99, 101, 102].

Device engineering using colloidal perovskite nanocrystals encounters the same challenges as known for polycrystalline PeLEDs, where balanced charge injection, interfaces, heat management and outcoupling are crucial factors to achieve high device performances and longer operation times. The major difference in the case of perovskite NC is the film deposition, as the crystals are usually pre-formed, and the resulting film contains the stabilizing ligands. Avoiding agglomeration of the nanocrystals and controlling their size and shape necessitates the utilization of these ligands. The ligands usually are long alkyl chains molecules of fatty acids (e.g., oleic acid) that have an insulation hydrocarbon backbone and their conjugation to nanocrystals can impede charge injection at the device stage, however, approaches to enhance the electronic coupling between the nanocrystals by utilizing shorter alkyl chains or using p-conjugated linkers were reported [99, 101, 102]. Surface defects as a result of detached ligands due to their dynamic nature or vacancy of halide ions due to leaching effects act as nonradiative trap sites and have pronounced impact on nanocrystals, when compared to bulk perovskites, as the former have a larger proportion of surface atoms. The defects can be reduced by compositional engineering as well as ligand exploitation, as shown by Kim et al. who used guanidinium doping in formamidinium nanocrystals which leads to segregation of the guanidinium to the NC surface, stabilizing undercoordinated sites. In conjunction, they introduced a bromide vacancy healing agent as a ligand, achieving one of the highest EQEs (23.4%) thus far [100].

### Thin-film and device processing steps

There is a close correlation between the quality of the perovskite layer and its photophysical properties: highly crystalline small grains, uniform morphology, pinhole free layer, and proper interface with charge injection layers are crucial for achieving high-performance PeLEDs and to suppress nonradiative recombination events [56, 103, 104]. Owing to the superior processability of solution and solid phases of perovskite materials, films fabrication is possible with numerous techniques [105]. Although significant results have been achieved for small-area perovskite photovoltaics, more attempts should be devoted to improving these new technologies and amend their performance specially for large-scale perovskite deposition and pave the way for commercialization [52, 106]. The major issues with hybrid perovskite LEDs at the current stage of progress are material and thermal stability, which pose barriers on the transfer of perovskite technology for practical applications. The color stability i.e., color change with duration of operation under the constant voltage, likely due to ion migration and operational stability, is essentially required. In addition, the passivation of surface defects and interfacial stabilization are crucial in enhancing the performance of PeLED technologies.

### Spin coating

The solution-based spin coating method is the most used technique for PeLED fabrication due to its simplicity, high compatibility to subsequent device steps and reproducibility. In addition, spin coating leads to rapid evaporation of the solvent that promptly starts the perovskite crystals nucleation and at the same time offers the possibility to control the on-substrate crystallization kinetics of perovskites films [105]. Finally, perovskite thin films can improve radiative recombination due to constrained dimensions, whene compared to their thick film analogs [56]. There are two general ways for depositing polycrystalline perovskite thin films with spin coating technique. In the one-step spin coating approach, which most common method for high-efficiency PeLEDs, the perovskite precursor solution is prepared and dropped on the target substrates during or before spinning. Most of the solvent is eliminated by centrifugal force, and a final wet film is obtained that should be annealed to promote crystallization [61, 78]. Antisolvent method is an approach to wash away the remaining precursor solvents, and initiate rapid crystallization to obtain a homogenous perovskite layer [56]. For the two-step method, a lead halide film is initially deposited by spin coating and then converted to final perovskite by treatment with organic halide salts [78]. In spite of more complicated process for two-step deposition, it usually enables to fabricate more smooth and compact films that means less nonradiative recombination centers [78]. Although, spin-coating is still the most used method for PeLEDs fabrication and EQE for spin-coated PLEDs have reached > 20%, this method is undesirable for large-scale commercialization [78, 107]. Indeed, a significant amount of precursor ink is wasted during solution during spinning process that is likely to increase the fabrication costs and decrease the materials economy at larger scales. More importantly, circumferential speeds variation along the radial direction of the samples often results in different rate of perovskite crystal growth at the edges and center of the sample and is manifested in formation of non-uniform films that prevents upscaling production with this method [105, 108].

### Vapor phase deposition

**Fig. 9:**
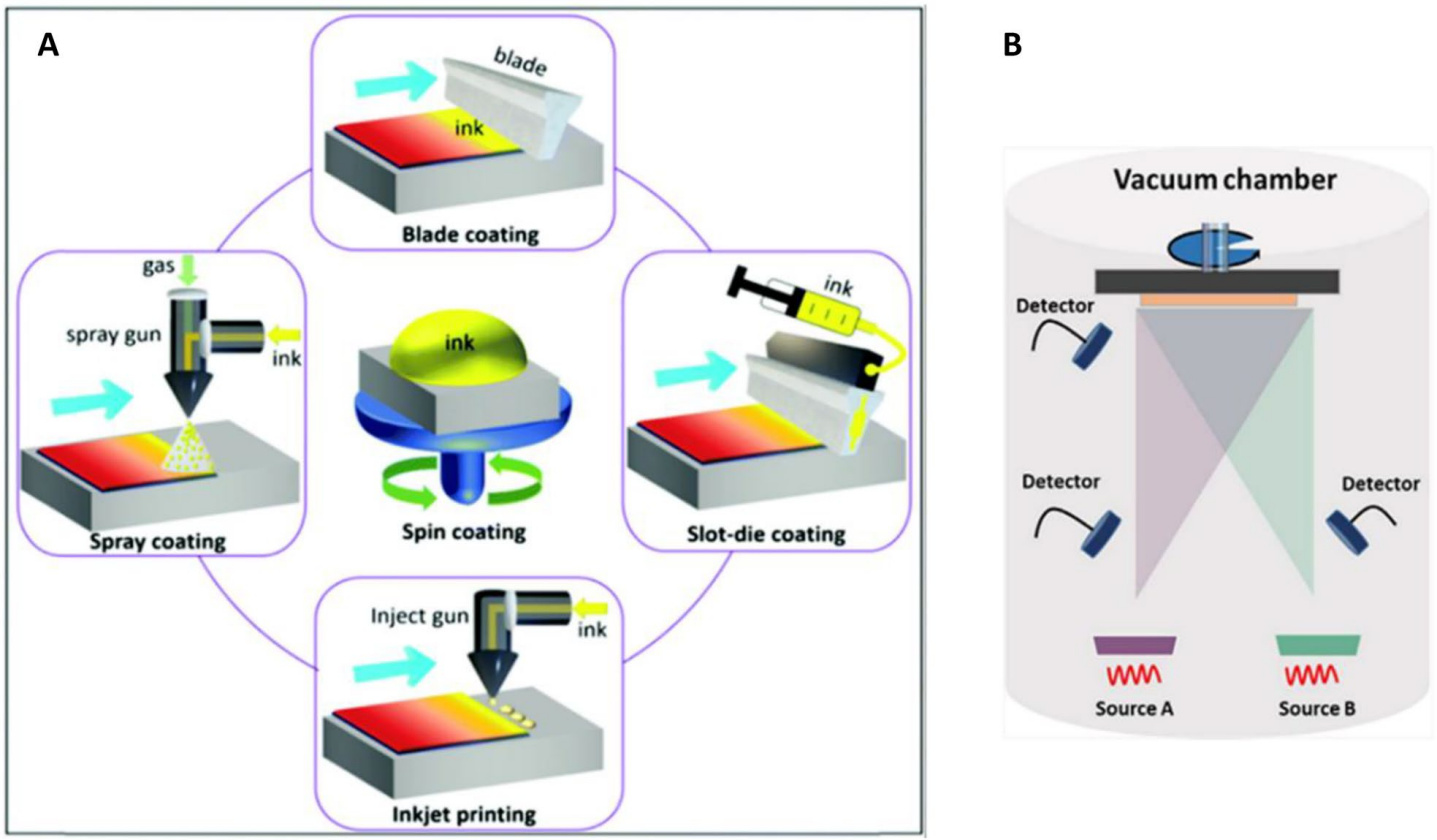
**a** Schematic illustration of perovskite deposition techniques. Reprinted from Ref. [52] Copyright © 2021 Energy and Environmental Science. **b** Vacuum thermal evaporation setup schematic illustration (Reprinted from Ref. [78] Copyright © 2021 The Author(s). Small Science published by Wiley–VCH GmbH)

The vapor deposition technologies, including chemical vapor deposition (CVD) and physical vapor deposition (PVD), developed for fabrication perovskite thin-films generally deliver higher quality films as the intermediate processing steps such as use of anti-solvents, annealing etc. can be curtailed. However, the perovskite film growth is generally done under high vacuum atmosphere, hence they are considered as vacuum thermal evaporation (VTE) methods (Fig. 9b) [27]. The VTE methods are promising choice to grow emissive layers, which is not affected by the low solubility of the perovskites. The VTE approaches have been developed as the most dominant process for industrial preparation of OLED displays. Therefore, the well-developed evaporating facilities and equipment could be adapted to fabricate PeLEDs [78]. The CVD process must be done in few elementary steps, involving the sublimation of solid precursors that can be sequentially evaporated or co-evaporated with heating under high vacuum condition. During the deposition step, the precursor vapors transport efficiently to the target substrate guided by the reduced pressure, to gradually deposit on the cooler substrate, which eliminates post-treatment annealing process and enabling the perovskite thin film deposition on flexible, soft, and even rough substrates [27, 78]. The vapor-phase deposition method possesses high potential to be used in large scale perovskite thin-film deposition, but often it is necessary to use expensive and complex vacuum equipment and process monitoring systems. Also, long processing times for finalizing the thin-film deposition seriously hinder the applications of VTE methods in low-cost and facile manufacturing of perovskite photovoltaics and light emitting diodes [109].

### Large scale production methods

To achieve highly emissive large-size perovskite films, a diversity of mass-production techniques such as spray-coating, blade-coating, ink-jet printing etc. have been investigated [107]. Given the recent progress and development of large-area PSCs, these approaches have been fostered for the large-area perovskite thin film growth, and it can provide a proper guideline for large-area PeLEDs emitting layer fabrication [27]. For large-scale production methods, the present challenges are to control the crystallinity of final layer, uniformity, film thickness and morphology [107].

#### Doctor blade coating

**Fig. 10:**
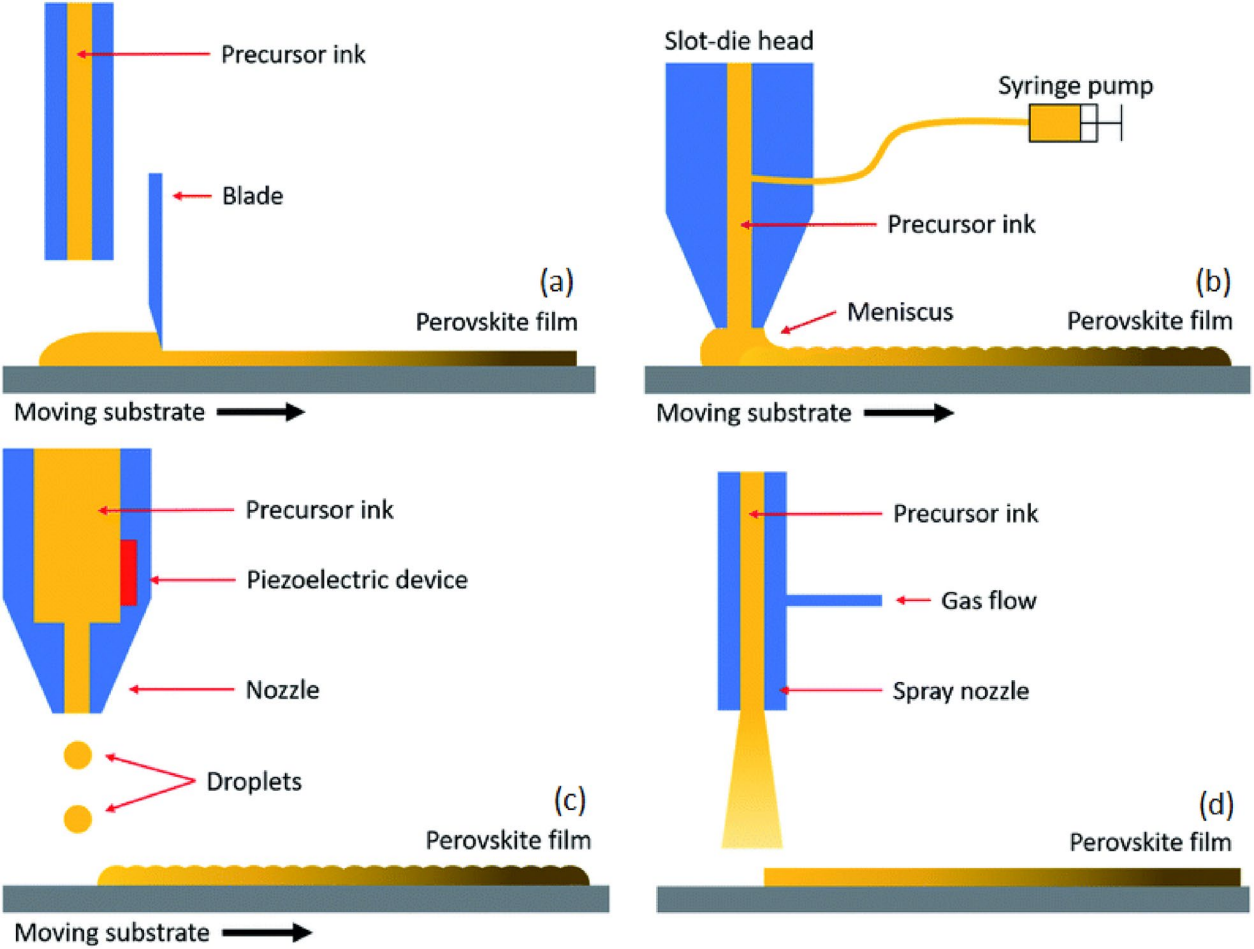
Schematic illustrations of **a** Doctor blade-coating, **b** slot-die coating, **c** inkjet printing, **d** spray coating (Reproduced from Ref. [111] with permission from the Royal Society of Chemistry)

The doctor blade coating (Fig. 10a) is one of the most promising meniscus coating methods for deposition of the perovskite films, while metering the amount of ink that needs to be left on the surface. In this method, a definite amount of ink is poured directly at edge of the blade which is held above the substrate surface and moved at a specific speed to control a specific amount of ink to stay on the substrate. The EQE of doctor-bladed PeLEDs reached > 16%, and the expected cost as low as 0.02 cent/cm^2^ of emissive layer indicates its application potential [78].

#### Slot-die coating

The concept of slot-die coating (Fig. 10b) represents a versatile deposition technique in which a solution or slurry is delivered onto a substrate via a narrow slit formed by two parallelly organized metal blades [52]. The slot-die coating enables uniform films across large area and offers a better parametric control between film thickness, the flow rate of perovskite ink and the movement speed of the coated substrate relative to the slot. A reservoir for precursor ink can impede the deterioration of the ink and leads to a higher quality and reproducibility of perovskite layer in comparison to doctor blade-coating in which the perovskite ink is exposed to air during process and thus prone to environmental degradation [27]. The film thickness during a slot-die process is adjustable by controlling the flow of ink pumped to the coating-head and the speed of the head [110].

#### Inkjet printing

Inkjet printing (Fig. 10c) is an industrially used technique, which generates microdroplets at a nozzle aperture, and transfer them on to the target substrate through piezoelectric stimulus. This well-controlled, non-contact printing process utilizes digital management technology and is indispensable in the printing industry [52]. The advantages of digital inkjet printing include its scalability, material and cost savings due to minimal waste of functional materials, and the ability to print on diverse substrates with arbitrary shapes [112]. Recently, there have been remarkable advancements in inkjet printing technology, particularly in the realm of perovskite quantum dot (QD) thin films. Wei et al. have achieved a significant breakthrough by fabricating high-quality printed perovskite QD thin films with an exceptional external quantum efficiency (EQE) of 8.54%. This groundbreaking achievement represents the best performance to date for inkjet-printed PeLEDs [68].

#### Spray coating

Spray coating (Fig. 10d) is a solution-based process where atomized droplets are ejected from the surface of a liquid and are directed by pressured carrier gas onto the target surface to form a wet film. In the final stage, the ink solvent is evaporating and the target material is crystallized as the ink solvent vaporizes [52, 113]. Controlling the crystallization kinetics is a critical aspect of the spray coating process, typically achieved through substrate temperature monitoring or post-deposition annealing steps. These techniques allow researchers to fine-tune the material’s crystalline structure and optimize its properties for specific applications. Several key parameters significantly influence the final film properties, including the precursor concentration in the solution, uniformity, and size of the droplets, carrier gas pressure, and the drying speed [27]. Precise control of these factors is essential to achieve the desired film thickness and morphology [27].

## Device stack and resulting efficiency

While the PLQY of perovskites is an important material property to determine the feasibility of its usage in light emitting devices, the efficiency of such devices is dependent on a range of materials characteristics and device parameters. A prime example for this intricate interplay is evident in the difference between the PLQY of perovskite nanocrystals in solution, which are often around 90% and the PLQY of nanocrystal thin films from the same batches, which show ~ 60% PLQY if no post-treatment is done [114, 115]. In addition, when transitioning to scalable manufacturing methods, the precursor inks have to be totally redesigned as different physical conditions for coating and solidification apply to scalable solution-processing techniques such as slot-die coating and inkjet printing. Moreover, the device structure, fabrication, choice of electrodes and possible adjacent charge injection layers contribute to the total efficiency and luminance output of the fully assembled device. Modulating the interface between the perovskite layer and the hole-transport and electron-transport layers (HTL and ETL) could considerably optimize the overall efficiency. Thus far, the highest luminance was achieved with green emitting PeLEDs, while the EQE of green, red and near-infrared PeLEDs all exceeded 20% [11, 14, 116, 117]. Interestingly, the tabulated perovskite layers tested in PeLED devices were all prepared from solution-based techniques. Generally, current–voltage-luminance data is gathered in individual cases from which the EQE and luminous efficacy can be calculated. The information on the efficiency of PeLEDs has to be taken with a grain of salt, as no standard certification protocol exists so far [118]. The following Table 1 provides a summary of device performance of PeLEDs developed in recent years.

**Table 1:** Perovskite composition and device characteristics of the best performing thin film PeLEDs for blue, green, red and near-infrared light

Color	Material	EL (nm)	Luminance (cd/m^2^)	Device structure	EQE (%)	References
Blue	(L-PMexPEA1-x)2Csn-1Pbn(ClyBr1-y)3n + 1	497	1000	ITO/PEDOT:PSS/perovskite/TPBi/LiF/Al	12.43	[119]
Blue	PEA_2_(Cs_1-*x*_EA_*x*_PbBr_3_)_2_PbBr_4_	488	–	ITO/m-PEDOT:PSS/Perovskite/TBPi/LiF/Al	12.1	[70]
Blue	CsPbBr_3_:PEACl:YCl_3_ (quasi 2D)	485	9040	ITO/PEDOT:PSS/perovskite/TPBi/LiF/Al	11.0	[120]
Blue	(PBABr):(Cs/FA/MA)Br:PbBr_2_	493	1151	ITO/PEDOT:PSS/Q2DPe/POT2T/LiF/Al	5.08	[121]
Blue	(PEA:NPA)Cs_*n*−1_Pb_*n*_Br_3*n*+1_	485	1200	ITO/PVK/Q2DPe/PO-T2T/Liq/Al	2.62	[122]
Blue	BA_2_DMA_1.6_Cs_2_Pb_3_Br_11.6_	490	2825	ITO/PEDOT:PSS or NiO_*x*_/Q2DPe/TPBi/LiF/Al	2.4	[123]
Blue	DPPOCl treated PEA_2_Cs_1.6_MA_0.4_Pb_3_Br_10_	489	5141	ITO/PEDOT:PSS:PFI/Q2DPes/TPBi/LiF/Al	1.3	[124]
Blue	CsPbBr_3_/PbBr_*y*_ (or PbCl_*y*_) (core/shell) QDs	508	2880	ITO/ZnO/CsPbBr_3-x_Cl_x_/PVK/V_2_O_5-δ_/C/Pt/Al	0.72	[125]
Green	PEA_2_(FAPbBr_3_)_2_PbBr_4_	524	6514	ITO/Ni_0.9_Mg_0.1_O_x_/PVK/PEG/ Perovskite/TPBi/LiF/Al	30.84	[126]
Green	CsPbBr_3_/MABr (Quasi-core/shell 3D)	525	14,000	ITO/PEDOT:PSS/perovskite/PMMA/B3PYMPM/LiF/Al	20.3	[116]
Green	BA_2_Cs_4_Pb_5_Br_16_	517	6785	ITO/TFB:PVK/perovskite/LiF/TPBi/LiF/Al	16	[69]
Green	CsPbBr_3_-PEABr	510	45,000	ITO/PVK/Perovskite/TPBi/LiF/Al	15.3	[127]
Green	(Cs_1−*x*_Rb_*x*_)_1−*y*_K_*y*_PbBr_3_	520	156 155	LiF/perovskite/LiF/ZnS/ZnSe	11.05	[14]
Green	(PEA)_2_FA_2_Pb_3_Br_10_	520	41 500	ITO/PEDOT:PSS/Perovskite/TPBi/LiF/Al	10.6	[74]
Green	MAPbBr_3_ QDs:PVP	525	43,990	ITO/PEDOT:PSS/PSK/In-Ga	10.5	[128]
Green	CsPbBr_3_	531	22,298	ITO/PEDOT:PSS:PFI/ Perovskite/TPBi/LiF/Al	9.64	[129]
Green	PEA_2_(FA_0.5_Cs_0.5_)_*n*−1_Pb_*n*_Br_3*n*+1_	520	45,600	ITO/PEDOT:PSS/Perovskite/TPBi/LiF/Al	5.9	[130]
Green	MAPbBr_3_ 3D/2D		–	FTO/Buf-HIL/MAPbBr3/TPBi/LiF/Al	4.23	[76]
Green	MAPb(Cl/Br/I)_3_	524	1861	ITO/PEDOT:PSS/Perovskite/TPBi/LiF/Al	2.65	[131]
Green	CsPbBr_3_	521	1008	ITO/PEDOT:PSS/CsPbBr_3_/ZnO/Ag	0.14	[132]
Green	CsPbBr_3_	518	2.5	ITO/PEDOT:PSS/Poly-TPD/Perovskite/ZnO/LiF/Al	0.002	[133]
Red	(PEA/m-F-PEA)_*x*_NMA_1−*x*_)_2_CsPb_2_I_7_	680	1300	ITO/m-PEDOT:PSS)/poly-TPD/PVK/perovskite/TPBi LiF/Al	25.8	[134]
Red	CsPbI_3_ (QD)	640	1000	ITO/PEDOT:PSS/Poly-TPD/Perovskite/MoO_X_/Ag	23	[11]
Red	CsPb(Br/I)_3_ (QD)	653	10.3	ITO/PEDOT:PSS/poly-TPD/perovskite/TPBi/Liq/Al	21.3	[135]
Red	CsPbI_3_ nanowires	600	13,644	ITO/PEDOT: PSS/PVK/Perovskite/ZnO/Ag	6.5	[84]
Red	CsPbI_3_	683	–	FTO/TiO_2_/CsPbI_3_/PTAA/MoO_3_/Ag	3.8	[136]
Near-IR	ODEA-FAPbI_3_ (3D)	800	–	ITO/ZnO:PEIE/perovskite/TPBi/MoO_3_/Au	21.6	[117]
Near-IR	CH_3_NH_3_PbBr_3_ single crystal	799	–	ITO/ZnO/PEIE/FAPbI_3_/poly-TPD/MoO_3_/Al	20.2	[10]
Near-IR	FPMAI-MAPb0.6Sn_0.4_I_3_	917	–	ITO/PolyTPD/perovskite/TPBi/LiF/Al	5	[137]

### Colloidal perovskite nanocrystals devices

As the perovskite nanocrystals are usually preformed, the subsequent deposition of a dense, pinhole free layer is challenging, however, the separation of synthesis and deposition steps allow for more control over the crystallization and as such quality, uniformity and properties of the individual crystallites. The most common methods for deposition of perovskite nanocrystals include spin coating, blade coating, inkjet printing or bar-coating [138–141]. The recently reported bar-coated PNC films exhibited an EQE of 23.26% on a small-size sample (4 mm^2^ pixel size), whereas a large-scale device (102 mm^2^) was found to have an EQE of 22.5% [142]. This methodology appears promising for large-scale roll-to-roll fabrication for PeLEDs, making it a favorable option for industrialization of perovskite technologies. An additional step towards commercialization was achieved by splitting a bulk perovskite layer into nanocrystals with benzyl phosphonic acid, combining the ease of perovskite film preparation with the advantages of perovskite nanocrystals, resulting in a record EQE of 28.9% [141] (Table 2).

**Table 2:** Selected examples of perovskite nanocrystal-based LED parameters achieved by different engineering strategies

Material	Strategy	Luminance (cd/m^2^)	EQE (%)	References
FA_0.7_MA_0.1_GA_0.2_)_0.87_Cs_0.13_PbBr_3_	On substrate nanocrystal synthesis	470,000	28.9	[141]
FA_1−*x*_GA_*x*_PbBr_3_	NC defect passivation	–	23.4	[100]
MAPbBr_3_	MOF stabilized	120,000	15.9	[97, 143]
CsPb_0.64_Zn_0.36_I_3_	B-site cation variation (Zn^2+^)	2202	15.1	[97]
CsPbI_3_	Inorganic ligand	–	23	[11]
Cs_1−*x*_GA_*x*_PbI_3_	A cite cation engineering	3486	18.9	[144]
CsPb(Br/I)_3_	In situ modification	11,233	13.2	[145]
CsPbBr_3_:F	Fluoride post-synthesis treatment	1946	16.9	[146]
CsPbI_3_	Multidentate short ligand tetramethylthiuram disulfide (TMTD) process	3861	20.65	[147]

**Fig. 11:**
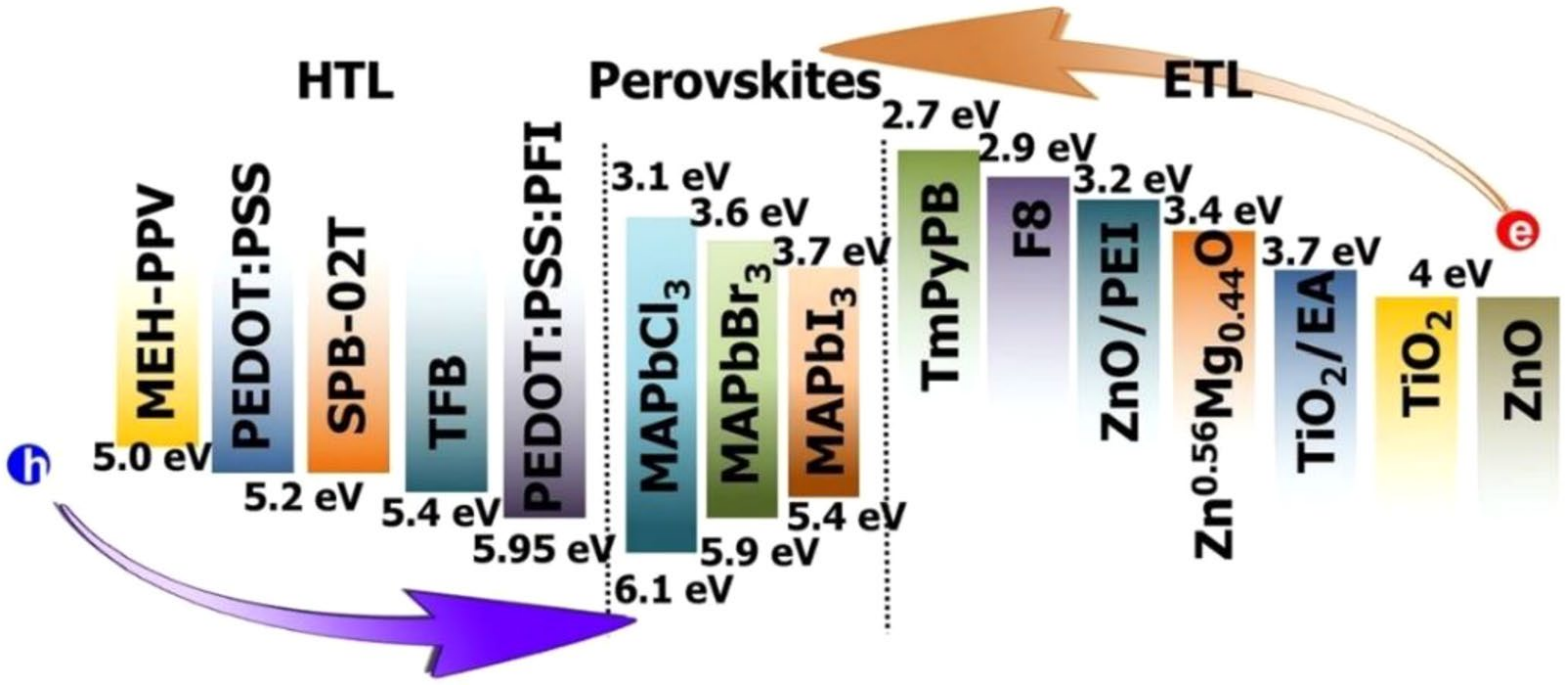
Energy band diagram for common ETL and HTL in perovskite light emitting diodes (Reprinted from Ref. [154] Copyright © 2016 National Academy of Sciences)

It is noted that most of the well performing PeLEDs are built on ITO/poly-3,4-ethylenedioxythiophen:polystyrene sulfonate (PEDOT:PSS) and utilizing aluminium (Al) as the cathode. Specific modification in HTL, ETL or perovskite layers were demonstrated to modify the emission wavelength and corresponding luminance color [148]. Also, additional passivation or interlayers were introduced in between to improve the device performance and stability [149]. For example, the conventional PEDOT:PSS was doped with perfluorinated ionomer (PFI) for improved exciton-buffering between the HTL and perovskite layers [124]. Other efforts includes the utilization of TFB, PVK, poly-TPD, NiO_X_¸ etc. in discrete and combinational strategies which appear to deliver potentially competitive HTL materials for PeLEDs [69, 127, 137]. The inclusion of NiO_X_ film is noted to advance highly crystalline perovskite growth by reducing the trap density of states compared to the conventional PEDOT:PSS, which leads to improved optical properties, photostability of the perovskite/HTL interface and overall stability of the device [150]. 1,3,5-tris(1-phenyl-1Hbenzimidazol-2-yl)benzene (TPBi) is the most commonly used ETL for appropriate band alignment and efficient electron injection properties [10, 11, 69, 116, 122, 127, 135, 137, 151]. As an alternative organic ETL, 2,4,6-tris(3-(diphenylphosphoryl)phenyl)triazine (POT2T) is also employed to deliver impressive electron transport properties [122]. In some reports, the inorganic metal oxides, such as ZnO and MoO_3_ have also been used as alternative ETLs. The MoO_3_ interlayer adopts a similar function of LiF for Al electrodes, improving the charge injection [152]. In such cases, polyethylenimine (PEIE) or PTAA is sometimes utilized as blocking interlayer between metal oxide and perovskite to prevent charge accumulation and interfacial reactions and is proposed to lead to better crystallization [10]. Low work function (WF) metals like Ca and Mg are reactive in air, while the work functions of Al and Ag are too high for efficient injection, thus, a LiF interlayer is commonly utilized for better alignment of the HOMO or VB of the ETL materials and the work function of the metals [153]. In an alternative approach to reduce the WF around the cathode, lithium 8-quinolate (Liq) has also been demonstrated to deliver no loss in the performance [122, 135]. The deviating choice of electrode (Au) of the near-infrared emitting device is attributed to the inverse n-i-p stack. The enhancement in device efficiency for the halide perovskite-based LEDs is seen to be critically dependent upon the stack structure, and novel transport materials, interlayers and design strategies should be continuedly pursued. The energy band diagram for common ETL, HTL and CH_3_NH_3_PbX_3_ perovskites are shown in (Fig. 11).

## Challenges

### Perovskite stability in solid state

The limited stability of hybrid halide perovskites under ambient conditions are due to both intrinsic and extrinsic reasons. Whereas the occurrence of surface inhomogeneities and presence of interstitial defects and ionic migration contribute to intrinsic defects, the influence of environmental factors leading to chemical degradation are extrinsic factors, which need to be circumvented to make stable devices based on perovskite emitter films. While PeLEDs have a comparable EQE to the other available technologies, for commercialization of lead-based perovskite LEDs the main hurdle remains the operational stability. As over 10,000 h operational lifetime are needed for commercialization and only a few hundred hours were achieved so far, this issue remains as the most important challenge for researchers in the field of PeLEDs [22].

It is obvious, that the perovskite can quickly degrade, especially under increased temperature when considering the simple reactions:

2 $${\text{CH}}_{3} {\text{NH}}_{3} {\text{PbI}}_{3} \overset {} \rightleftharpoons {\text{PbI}}_{2} + {\text{CH}}_{3} {\text{NH}}_{3} {\text{I}}$$

3 $${\text{CH}}_{3} {\text{NH}}_{3} {\text{I}}\overset {} \rightleftharpoons {\text{CH}}_{3} {\text{NH}}_{2} + {\text{HI}}$$

4 $${\text{4HI}} + {\text{O}}_{2} \overset {} \rightleftharpoons 2{\text{I}}_{2} + {\text{2H}}_{2} {\text{O}}$$

5 $${\text{2HI}}\overset {} \rightleftharpoons {\text{H}}_{2} + {\text{I}}_{2}$$

As some of the reaction products are volatile and thus drive the reaction to the entropic side while rendering the degradation irreversible. Furthermore, each component within the chain of reactions can react with other compounds, such as electron or hole transporting materials in a perovskite device or even the electrodes, removing them from the equation and further drive the degradation of the perovskite.

The bias and current applied during operation of perovskite devices leads to two phenomena that are commonly named as the reasons for the fast degradation of the perovskite: ion migration and joule heating. While certain ion movements are reversible [155], the ions can migrate to the adjacent device layers and react with them at the interfaces, leading to even faster device degradation [156, 157]. The recent studies, insights and possible countermeasures regarding ion migration are examined in the following.

### Ion migration

**Fig. 12:**
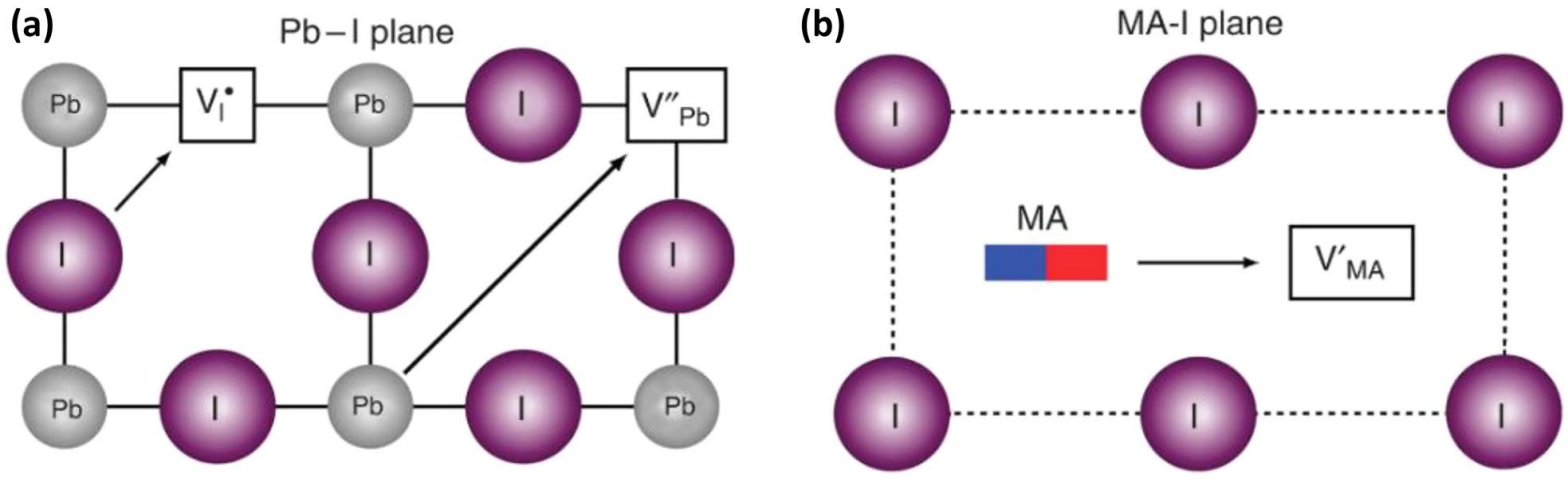
Defect site driven ion migration in CH_3_NH_3_PbI_3_ (Reprinted from Ref. [162] Copyright © 2015. The Author(s))

One of the main challenges in the growing field of perovskite solar cells and light emitting diodes is the limited operational stability of the photoactive layer. Among various factors, the mobility of ions is a possible reason for low stability, however underlying origin and mechanisms of transport remain unclear and a matter of debate. The voltage bias during operation apparently increases the ion migration rate and ultimately leads to destruction of the perovskite functionality manifested in the device degradation. The mobility of ions in lead-halide perovskites was discovered as early as 1987 [158] and numerous studies were conducted to determine the effect of ion diffusion in the materials [159, 160]. It is obvious that compared to photovoltaic application, the ion migration is even more pronounced in light emitting devices as a higher electric field is applied during operation [161]. Ion migration can be induced by heat or light and is reported to be defect site driven leading to degradation of metal electrodes in perovskite devices and phase change within the perovskites (Fig. 12) [162–165].

**Fig. 13:**
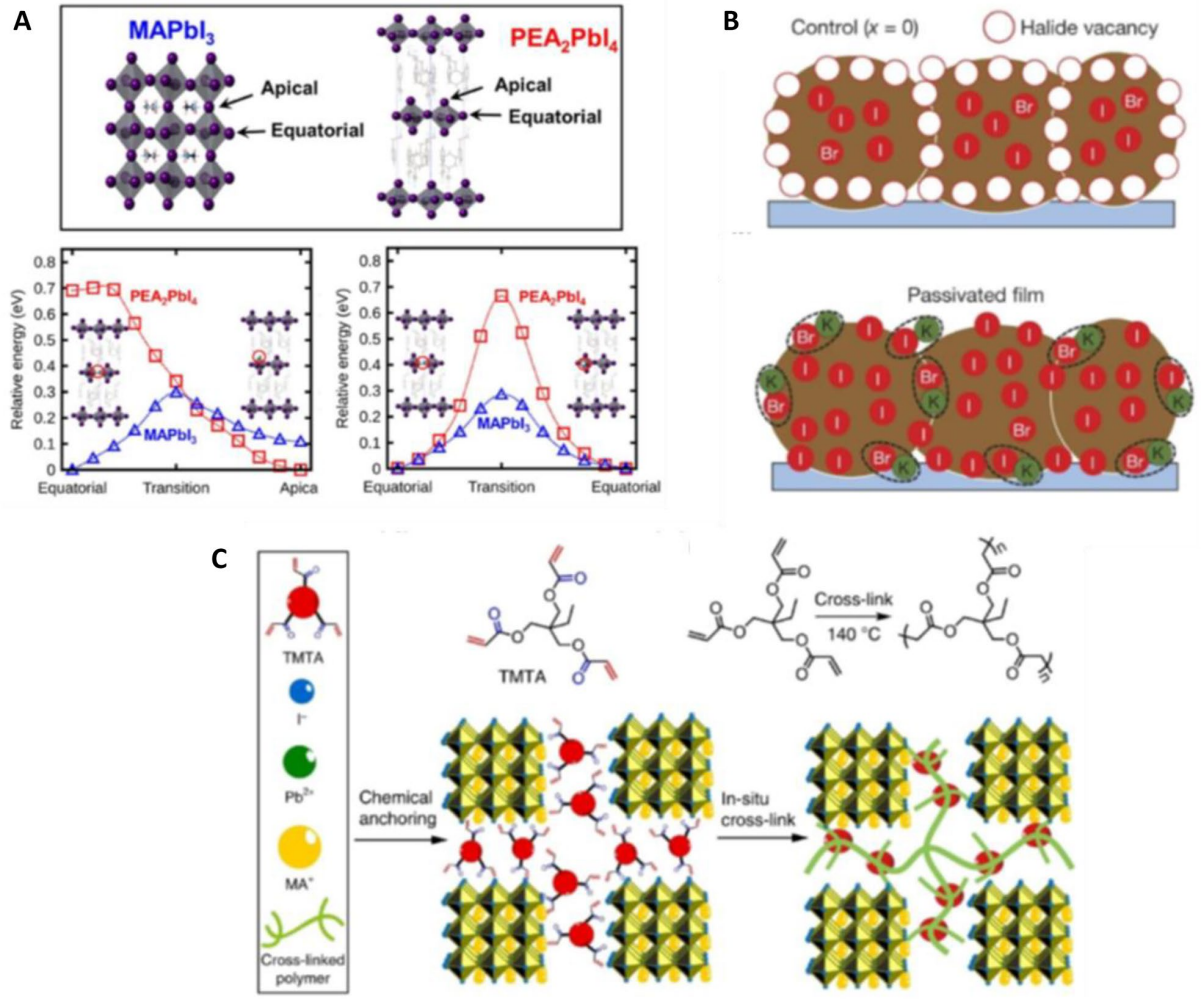
Strategies to reduce ion migration (exemplary). **a** Energetics of iodide vacancies in 3D MAPbI_3_ and 2D PEA_2_PbI_4_ during different migration paths using DFT calculations. Reprinted from Ref. [182] Copyright © 2017, American Chemical Society. **b** passivation with alkali cations with the example of KI in a perovskite film Reprinted from Ref. [173]. Copyright © 2018, Macmillan Publishers Limited, part of Springer Nature. All rights reserved. **c** Working mechanism of grain boundary passivation by organic agent, in this case with TMTA (Reprinted from Ref. [183]. Copyright © 2018, The Author(s))

One approach to suppress the mobility of ions is to grow highly crystalline films to reduce the defects within the perovskite. Polycrystalline perovskite exhibits two to three orders of magnitude higher trap densities than their high-quality single crystalline counterparts [166]. However, even in single crystalline perovskites, the energy necessary for ion movement are low, as Mingming et al. observed ion migration under an external electrical field as low as 0.3 V/μm even in single crystalline CH_3_NH_3_PbBr_3_ micro-platelets [167]. As the applied voltages to drive the LEDs are usually 2–10 V [5, 168] and the perovskite thin films have a thickness of a few nm to a few μm, the ion migration would not be avoided even with single crystalline films. All-inorganic perovskites were found to have a higher ion migration barrier as an influence of methylammonium was found by McGovern et al*.* They suggest to either use Cs^+^ as A-site cation or tailor the Pb:X stoichiometry to reduce the vacancies and thereby mitigate ion migration [169]. Zhang et al. engineered a fully inorganic (Cs_0.83_Rb_0.17_)_0.95_K_0.05_PbBr_3_ perovskite and achieved ~ 255 h of operational lifetime [14]. The inclusion of a small amount of alkali ions (through LiI, NaI, KI, RbI and CsI) was found to be helpful to reduce hysteresis in CH_3_NH_3_PbI_3_ and CH(NH_2_)_2_PbI_3_ perovskites with KI having the most advantageous effect [170]. Other studies revealed the occupancy is dependent on the ion size of the alkali ion and interstitial occupancy raises the ion migration energy barrier [171, 172]. Abdi-Jalebi et al. report utilization of a K-X additive only passivated the perovskite surfaces at the grain boundaries by donating halides, but K^+^ is not included in the perovskite structure (Fig. 13) [173].

Furthermore, lower dimensional perovskites are reported to suppress the ionic migration. At 260 K the total conductivity of 3D CH_3_NH_3_PbI_3_ is mainly driven by ionic conductivity with activation energy of 0.19 eV in the dark and 0.03 eV under 0.25 sun illumination while the 2D perovskite (BA)_2_(MA)_3_Pb_4_I_13_ did not show a transition from electronic to ionic conductivity even at 330 K regardless of light irradiation and had a constant activation energy of ~ 41 meV which is said to be in the range for electronic conduction [174]. Polymer additives as a barrier between perovskite grains were found to increase the stability of under continuous light irradiation, and the resulting perovskite solar cells can withstand higher voltages (~ 2 V reverse bias instead of 0.5 V for non-additive devices) [175]. Although the lower dimensional- and polymer passivated perovskites are promising regarding ion migration suppression, the charge separation (PV) or charge injection (LED) are usually hindered compared to pure or 3D perovskites [176, 177]. The electrical conductivity can be improved to a certain degree by utilization of dopants [178] or specifically tailored organic spacer molecules [177, 179]. Noteworthy, the interfaces between perovskite and adjacent layers can also be tuned and exploited for ion migration suppression. As an example, the PCBM in inverted-stack perovskite solar cells reduces ion migration by passivation of I^−^ defects on the perovskite surface and blocking grain boundary channels [180, 181].

### Phase segregation

**Fig. 14:**
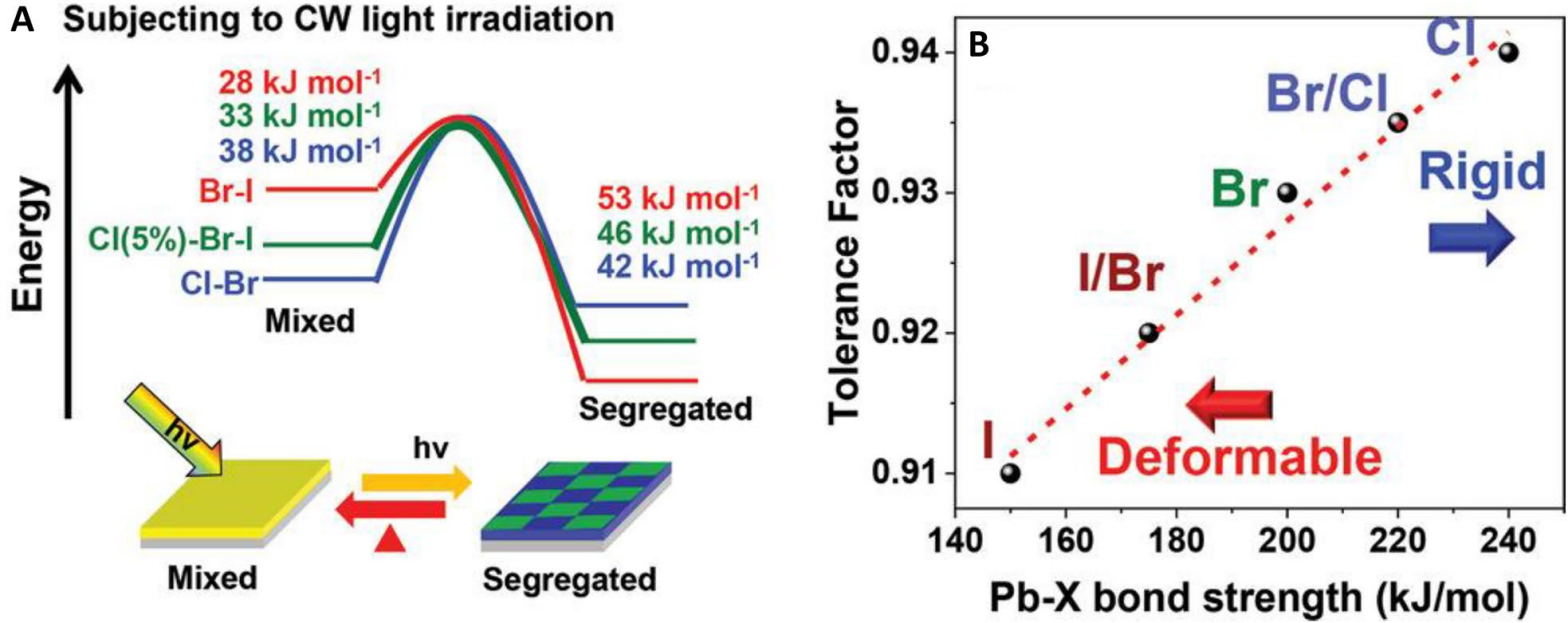
**a** Energetic diagram of ion migration in mixed halide perovskites leading to phase segregation under light irradiation and **b** bond strength-tolerance factor plot of Pb-X in lead-halide perovskites (Reprinted from Ref. [184]. © 2020 Wiley–VCH GmbH)

In the case of mixed halide perovskites, phase segregation can result from the high ionic mobility of the anionic species within the perovskite films. Inherent segregation (heterogeneous crystallization and growth) was observed by Cho and Kamat, when they tried to prepare CH_3_NH_3_PbI_1.5_Cl_1.5_ which possibly occurred due to the lattice mismatch of Cl and I based perovskites (Fig. 14) [184]. For non-inherently segregated, mixed halide-perovskites reversible segregation was observed by PL and XRD after short light illumination and subsequent regeneration of the initial compound after storage in the dark [163]. The stimuli can differ as shown by Zhang et al., who observed bias induced phase segregation in the dark [185]. The lattice mismatch between the different lead-halide perovskites reduces the activation energy of the migration step [169]. Tiede and coworkers found that defect density and iodide segregation correlate [186]. As such, obviously, the defect passivation strategies discussed for ion migration can also be applied to suppress phase segregation. Muscarella et al. found that application of pressure (0.3 GPa) to CH_3_NH_3_Pb(Br_x_I_1−x_)_3_ reduces phase segregation. They state, the same effect can be achieved by incorporation of smaller A-site cations [187]. Knight et al. studied the influence of A-site cations on the phase segregation of mixed-halide perovskites and noticed, MAPb(Br_0.5_I_0.5_)_3_ has a remarkably lower migration barrier than FA_0.83_Cs_0.17_Pb(Br_0.4_I_0.6_)_3_. They further found that after prolonged illumination, the ionic rearrangement could also be induced in the mixed A-site perovskite, possibly due to initial A-site demixing, however the A-site phase segregation is less studied [188]. MA^+^ electromigration is even responsible for a switchable photovoltaic effect [189]. For the mixed cation FA_x_MA_1-x_PbI_3_ infrared photothermal heterodyne imaging revealed spatial cation distributions with ~ 20% deviation on average, however the segregation occurred most likely in the fabrication stage [190].

### Joule heating

**Fig. 15:**
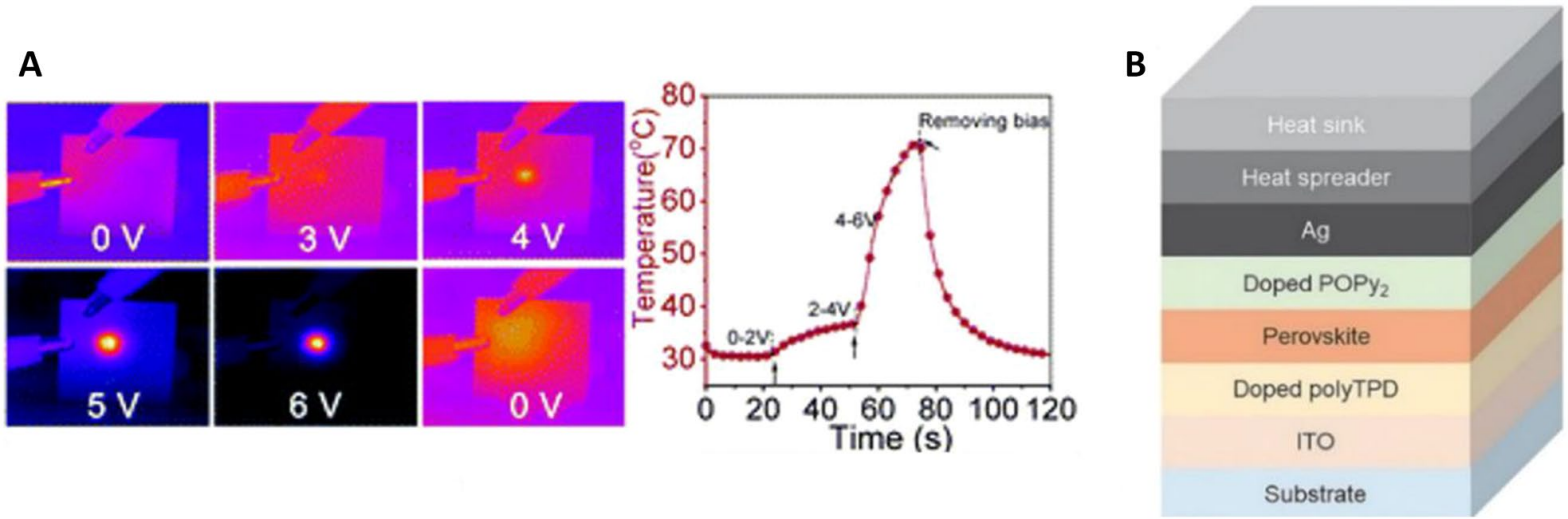
**a** Joule heating as recorded by a near-infrared camera viewed from ITO/glass surface under bias of 0–6 V and temperature–time plot. Reproduced from Ref. [127] with permission from the Royal Society of Chemistry. **b** Combination of heat sink with heat spreader as effective countermeasures to thermal degradation (Reproduced from Ref. [193] with permission. Copyright © 2020 WILEY–VCH Verlag GmbH & Co. KGaA, Weinheim)

As mentioned before, the operational stability of perovskite light emitting diodes remains as the major obstacle towards competition with the traditional III–V semiconductor and organic LEDs. While the operational lifetime can be improved to some degree, substantial breakthroughs are still waiting to be made. Joule heating, generation of heat when current flows through a conductor, when operating the device can heat the perovskites to well over 80 °C in under a minute [11]. This is especially concerning regarding the fact, that 90 °C are sufficient to release volatiles from CH_3_NH_3_PbX_3_ perovskites [191]. Qiu et al. noticed improved stability when thinner perovskite layers were utilized in their LEDs [137]. It was also found, that a high current operation leads to high energy side tails in the electroluminescence photon flux [192]. The conclusion from these two observations was that higher thickness (as well as a higher current) of the active layer leads to more heat which promotes thermally activated degradation such as electrochemical/redox reactions, acid/base reactions and photolysis [137, 192]. Their hypothesis was experimentally tested by implementing a carbon heat spreader and copper heat sink on the Ag top electrode, resulting in longer operational lifetimes as the temperature of the devices was lowered. Furthermore, lowering the active area of the device also resulted in less heat and thus longer lifetimes [193]. The thermal degradation is not limited to the perovskite active layer, but also affects the interfaces and other layers of the device stack, such as the commonly exploited transport material TPBi and reactive electrode materials such as Al and Ag [127, 194]. Potentially, high thermal conductive substrates could be utilized for more efficient passive cooling and in combination with heat spreader and heat sink as shown in (Fig. 15) as well as optimized active area and perovskite layer thickness would lead to much more stable devices.

The observations made by researchers from different fields suggest multiple factors are leading to similar detrimental effects in optoelectronic perovskite devices. Ion migration is gaining increased attention as the activation energies are low and the commonly utilized polycrystalline thin films are prone to contain high densities of mobile ions and vacancies as driving force. The resulting ion diffusion not only destroys the active layer, but also leads to reactions with adjacent layers and electrodes, increasing the electrical resistance of the device thereby increasing thermal stress through joule heating which in turn enables or increases degradation. One of the main advantages of perovskites making them especially interesting for tandem solar cells and light emitting diodes—the highly tunable bandgap, which enables full coverage of the visible electromagnetic spectrum—remains as challenging as the mixed halide phase segregation cannot easily be mitigated. Stoichiometry, crystallinity, reducing traps or trap passivation and mixed A-site cations are feasible strategies but require sophisticated equipment and reduce the facile perovskite device preparation which is one of the main advantages of these materials. Strategies for ion migration suppression and cooling of the perovskite devices are presented and should be combined for increased operational lifetimes.

## Conclusions

Considering the efforts reviewed and discussed here, the opportunities for perovskite LED technology are manifold. Their low-cost processability, abundant starting materials and superior (compositional) flexibility are a definite advantage over III–V semiconductors and organic LEDs. In only 5 years, the external quantum efficiency of PeLEDs was pushed to the current state-of-the art LED technologies, while the low operational stability remains as the most persistent and challenging task for researchers. The outstanding advantage in perovskite LEDs is the possible use of solution-based techniques at low temperatures such as spraying, doctor-blading and roll-to-roll printing, PeLEDs promise highly-efficient, light-weight and low-cost options leading to reasonable energy pay-back times. Pre-synthesizing high PLQY nanocrystals requires extra steps and the resulting layers after spin-coating are usually very thin and rich in pinholes. The most utilized perovskite deposition method is spin coating, which is facile, requires relatively cost-effective equipment, but is not suitable for large-scale fabrication and additionally most of the perovskite precursor ink is wasted during the spinning process. Vapor deposition methods have a high potential for large-area fabrication and high-quality materials, however come with the disadvantage of costly equipment. Cost-effective large-scale fabrication can be achieved by ink-jet printing, slot die coating or blade-coating, although the homogeneity of the resulting layers must be improved. The electron and hole injection layers heavily influence the efficiency in similar designed stacks as charge injection balance reduces power consumption and is less destructive towards the active material. External outcoupling strategies like lenses on top of the device for efficient light outcoupling and thermal management are often not included when selecting a stack for a device.

The major challenge faced by the hybrid perovskite LEDs at the current stage of progress relate to materials stability under passive (ambient storage) and active (device operation) environments, which pose barriers on the transfer of perovskite technology for practical applications. The color stability i.e. color change with duration of operation under a constant voltage, likely due to ion migration and operational stability, is an intrinsic limitation that calls for new chemical approaches to check the ionic transport. Furthermore, it is challenging to pinpoint the parameter that is determining the resulting efficiencies of the PeLEDs, as there is no “gold standard” for device stacks and measurement protocols. The most promising layers regarding the PeLED efficiency are ITO as substrate, PEDOT:PSS as HTM, TPBi or ZnO as ETM and LiF/Al as Electrode. Especially for electronically excited devices, ion migration remains one of the biggest challenges to overcome. The ion mobility is further driven by joule heating which by itself can be detrimental due to volatile compounds and temperature dependent phase transitions of the perovskite material. The analytical methods and understanding of the underlying mechanisms of ion migration still need to be developed further in order to find suitable and efficient countermeasures.

## Outlook

Perovskite research is not slowing down, as the versatility in application and composition-property relation continuously thrive the research interest. The review of the work on perovskite processing for perovskite devices revealed numerous studies focusing on the enhancement of properties, which indicates their potential in photovoltaic application, however the data also points out that in order to fulfill the promise of potential commercialization of perovskite solar cells or light emitting devices, some technical (e.g., operational stability) and environmental issues (e.g., metal toxicity, greener solvents) needs to be urgently addressed. Nevertheless, the spillover effect between the development in PV and PeLED materials is very synergistic. For example, some of the perovskite characteristics that are reported to be detrimental in PV devices, such as smaller grain size due to fast crystallization or additives, might prove useful for PeLED application as the charge carriers need to be confined for increasing the radiation recombination events. The measurement protocol for PeLEDs need to be standardized for better comparison of lifetime, luminance, and efficiency. The interdisciplinary interest in the material synthesis, its processing and modeling as well its implementation in devices promises huge potential for a fast development and should be exploited for tackling the current challenges. Finally, the application of data driven approaches in identifying the promising perovskite compositions act as the pointers for future growth in the field.

## Data Availability

Not applicable.

## Abbreviations

LEDs: Light emitting diodes
PeLEDs: Perovskite-based LEDs
PSCs: Perovskite solar cells
EQE: External quantum efficiency
ITO: Indium-doped tin oxide
FTO: Fluorine-doped tin oxide
HIL: Hole injection layer
EIL: Electron injection layer
HTL: Hole transport layer
ETL: Electron transport layer
EL: Emissive layer
LUMO: Lowest unoccupied molecular orbital
SWCNTs: Single walled carbon nanotubes
PET: Polyethylene-terephthalate
NWs: Nanowires
OLEDs: Organic light emitting diodes
FWHM: Full width at half maximum
PCE: Power conversion efficiency
NCP: Nanocrystal pinning
PL: Photoluminescence
FS: Femtosecond
BA: Butylammonium
PEA: Phenylethyl ammonium
NCs: Nanocrystals
QDs: Quantum dots
LARP: Ligand assisted reprecipitation
CVD: Chemical vapor deposition
PVD: Physical vapor deposition
VTE: Vacuum thermal evaporation
PEDOT:PSS: Poly-3,4-ethylendioxythiophen:Polystyrene sulfonfonate
PFI: Perfluorinated ionomer
TPBi: 1,3,5-Tris(1-phenyl-1Hbenzimidazol-2-yl)benzene
POT2T: 2,4,6-Tris(3-(diphenylphosphoryl)phenyl)triazine
PEIE: Polyethylenimine
WF: Work function
